# Biofluid-derived exosomes as next-generation biotherapeutic platforms to combat multidrug-resistant bacterial infections

**DOI:** 10.1093/rb/rbag137

**Published:** 2026-06-19

**Authors:** Anqi Liu, Haohang Xu, Yongfang Wang, Lingfeng Xu, Chao Liu, Hui Wang, Tong Ye, Xiaotong Sun, Linfei Chen, Xuan Wu, Xin Pang

**Affiliations:** School of Pharmacy, Henan University of Chinese Medicine, Zhengzhou 450006, China; College of Chemical Engineering, Fuzhou University, Fuzhou 350108, China; School of Pharmacy, Henan University of Chinese Medicine, Zhengzhou 450006, China; School of Pharmacy, Henan University of Chinese Medicine, Zhengzhou 450006, China; School of Pharmacy, Henan University of Chinese Medicine, Zhengzhou 450006, China; School of Pharmacy, Henan University of Chinese Medicine, Zhengzhou 450006, China; School of Pharmacy, Henan University of Chinese Medicine, Zhengzhou 450006, China; School of Pharmacy, Henan University of Chinese Medicine, Zhengzhou 450006, China; School of Pharmacy, Henan University of Chinese Medicine, Zhengzhou 450006, China; School of Pharmacy, Henan University of Chinese Medicine, Zhengzhou 450006, China; School of Pharmacy, Henan University of Chinese Medicine, Zhengzhou 450006, China

**Keywords:** exosomes, bacterial infection, combination therapy, drug delivery, immunoregulation

## Abstract

Bacterial infections pose a pervasive and escalating global health threat, exacerbated by the relentless emergence of multidrug-resistant pathogens. This crisis demands innovative therapeutic strategies that transcend traditional antibiotic paradigms. Within this context, exosomes, naturally secreted nanoscale extracellular vesicles, have emerged as compelling therapeutic alternatives and delivery vehicles. These endogenous phospholipid bilayer vesicles carry diverse bioactive cargo derived from their parent cells, facilitating direct antibacterial effects while actively orchestrating host immunomodulation through antigen presentation and immune response regulation. This multifaceted functionality positions exosomes as potent tools capable of not only eradicating pathogens but also modulating the complex host–pathogen interface and enhancing host defenses. This review systematically evaluates the burgeoning role of exosomes in combating bacterial infections. The fundamental methodologies for isolating, characterizing and classifying exosomes relevant to infectious disease research are summarized. Subsequently, we critically analyze their intrinsic antibacterial properties and explore advanced engineered therapeutic mechanisms. The discussion further examines innovative strategies integrating exosomes with complementary treatments and details their preclinical applications across diverse infection pathologies. Finally, we outline key translational challenges and future research directions essential for advancing the development of next-generation antibacterial exosomes with enhanced efficacy and reduced off-target toxicity.

## Graphical abstract

**Graphical Abstract:**
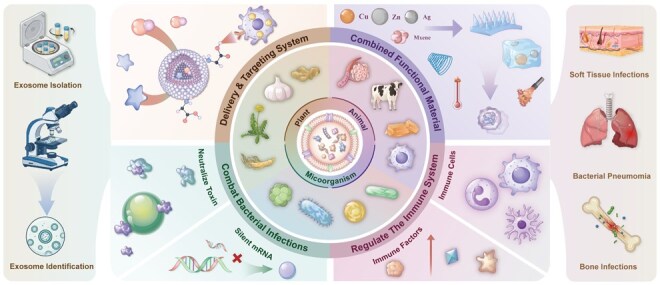

## Introduction

Bacterial infections remain a formidable and escalating threat to global public health, imposing a staggering burden on modern healthcare systems through persistent morbidity and mortality. While the mid-twentieth century was defined by the triumphant success of antimicrobial therapy, the efficacy of conventional antibiotics has been progressively undermined by the relentless evolution of multidrug-resistant (MDR) pathogens [1]. This crisis highlights a critical therapeutic impasse driven by the intrinsic limitations of current pharmacological interventions. Standard small-molecule treatments frequently suffer from suboptimal pharmacokinetic profiles and inadequate tissue penetration, which are particularly evident when addressing pathogens like methicillin-resistant *Staphylococcus aureus* or *Pseudomonas aeruginosa*. These bacteria can establish persistent intracellular reservoirs within host immune cells and form dense biofilms that act as physical and biochemical shields against antibiotic entry [2]. Furthermore, traditional antimicrobials typically focus on direct bactericidal effects while failing to modulate the dysregulated host immune response that often dictates the ultimate clinical outcome [3]. Consequently, there is an urgent and unmet need to transcend traditional antibiotic paradigms by developing innovative strategies that can simultaneously eradicate sequestered pathogens and restore immune homeostasis.

In the pursuit of next-generation anti-infective modalities, several biological and precision-based strategies have emerged as promising alternatives to small-molecule antibiotics [4]. Engineered monoclonal antibodies offer high-precision pathogen neutralization and toxin sequestration, while antimicrobial peptides (AMPs) provide rapid membrane-disrupting activity with a lower propensity for resistance development [5]. Additionally, the resurgence of phage therapy has demonstrated remarkable specificity in targeting recalcitrant bacterial strains without perturbing the commensal microbiota [6]. However, the clinical translation of these diverse agents is often hindered by shared biological barriers. For instance, antibodies and AMPs frequently face challenges related to systemic stability, potential immunogenicity and limited access to deep-seated infection sites or intracellular compartments. Phage therapy, while potent, remains constrained by narrow host ranges and the risk of rapid clearance by the host immune system [7, 8]. These collective limitations underscore the imperative to explore superior biological delivery systems that can stabilize these diverse payloads, facilitate deep tissue penetration and circumvent the innate barriers of the host microenvironment.

Amid this evolving therapeutic landscape, extracellular vesicles (EVs) have emerged as pivotal mediators of intercellular communication with profound implications for infectious disease biology. To ensure terminological and biological rigor, it is essential to delineate the distinct classes of EVs based on their biogenesis, which cross mammalian and non-mammalian kingdoms [9]. Classical exosomes represent a specific mammalian EV subpopulation (typically 30–150 nm) that uniquely originates from the endosomal system through the inward budding of multivesicular bodies and their subsequent fusion with the plasma membrane [10]. This complex mammalian biogenesis, regulated by endosomal sorting complexes required for transport-dependent or -independent pathways and molecular switches such as Rab11a and the Rubicon-WIPI2d axis, endows classical exosomes with specific tetraspanin markers (e.g. CD9, CD63 and CD81) [11]. In contrast, therapeutic platforms derived from non-mammalian sources possess distinct biogenetic routes: plant-derived exosome-like nanoparticles (ELNs) are assembled and secreted via specialized plant cellular pathways, while bacterial outer membrane vesicles (OMVs) are produced by the outward budding and pinching of the outer membrane of Gram-negative bacteria [12, 13]. Despite these distinct evolutionary and biogenetic origins, these nanoscale vesicles are collectively discussed under the broad umbrella of advanced vesicle-based therapeutics due to their shared structural architecture—an endogenous phospholipid bilayer that protects a diverse lumenal cargo, including proteins, lipids and various RNA species, from enzymatic degradation in harsh physiological environments [9]. This inherent versatility allows both classical exosomes and related cross-kingdom EVs to traverse stringent biological barriers, such as the blood–brain barrier, and facilitates the delivery of therapeutic payloads into otherwise inaccessible niches, including deep-seated biofilms and intracellular bacterial sanctuaries [14].

The rapidly expanding field of EV biology has illuminated the multifaceted roles these vesicles play as active participants in the dynamic “arms race” between host and pathogen [15]. During bacterial invasion, host-derived exosomes transcend their role as passive messengers to function as critical antimicrobial immune factors. They can orchestrate endogenous defense mechanisms by facilitating pathogen clearance, presenting antigens to prime immune surveillance and regulating inflammatory cascades through the horizontal transfer of bioactive molecules [16]. For instance, mesenchymal stem cell-derived exosomes (MSC-Exos) have demonstrated a remarkable capacity to reprogram the immune microenvironment by mitigating pro-inflammatory cytokines like TNF-α and IL-6 while promoting tissue repair, a synergy that is indispensable for managing sepsis and chronic diabetic wounds [17]. However, this biological dialogue is bidirectional and highly complex, as pathogens may exploit exosomal pathways to disseminate virulence factors or employ molecular mimicry to subvert host defenses. This dual nature positions exosomes at the nexus of infection pathogenesis and therapeutic intervention [18]. By leveraging their natural tropism or further enhancing it through surface engineering with ligands such as mannose or antimicrobial peptides, exosomes can be transformed into “smart” nanoplatforms for the targeted delivery of conventional antibiotics, nucleic acid-based regulators targeting resistance genes like mecA and other bioactive agents [19].

**Figure 1:**
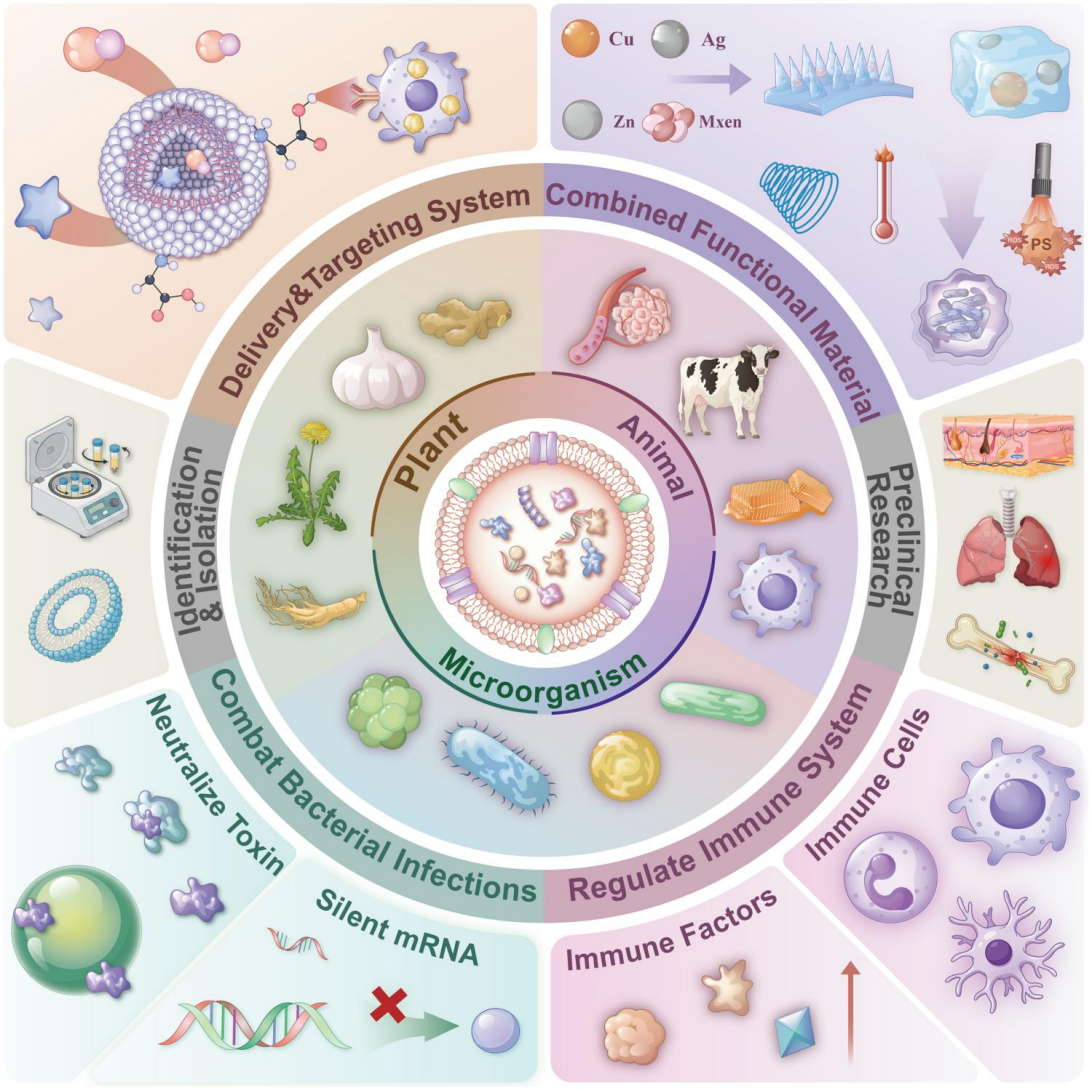
This figure illustrates the therapeutic potential of exosomes in combating bacterial infections. It systematically summarizes the diverse origins of exosomes (animal, plant and microbial), alongside their isolation and identification methods. Furthermore, it highlights their multifaceted antibacterial mechanisms, including direct bacterial clearance and immunomodulation. The schematic also emphasizes the innovative applications of exosomes as targeted nanodrug carriers and their integration with functional materials, concluding with an overview of their preclinical therapeutic efficacy across various infected tissues.

This review provides a comprehensive and critical synthesis of current advances in exosome research related to bacterial infections. We systematically examine the methodologies for vesicle isolation, characterization and the distinct molecular mechanisms governing the biogenesis and cargo sorting of mammalian exosomes, plant-derived ELNs and bacterial OMVs. Furthermore, we explore their intrinsic antibacterial properties, the latest engineering strategies for precision targeting and the integration of exosome-based delivery systems with existing clinical modalities. By synthesizing these multifaceted perspectives, this review aims to identify pivotal research gaps and illuminate translational pathways toward improving the diagnosis and management of recalcitrant bacterial infections (Figure 1). Ultimately, this cross-kingdom synthesis aims to construct a unified framework that bridges the biological distinctiveness of mammalian, plant and bacterial vesicles with their translational engineering principles. By doing so, this review seeks to inspire the development of adaptable, multifunctional nano-therapeutics capable of overcoming the multidrug resistance crisis in clinical settings.

## Isolation and identification of exosomes

The rigorous isolation and precise characterization of exosomes represent the cornerstone of extracellular vesicle research and are indispensable for elucidating their multifaceted biological functions or translating them into clinical applications. This critical process requires methodologies capable of selectively capturing these nanoscale vesicles while strictly preserving their structural integrity and bioactivity against a background of complex biological fluids [20]. A primary challenge in this field arises from the significant physicochemical overlap between exosomes and other extracellular entities, including apoptotic bodies, protein aggregates and lipoprotein particles. This complexity is further exacerbated in the context of infectious diseases where host-derived exosomes must be distinguished from bacterial OMVs or viral particles that share similar size ranges and sedimentation properties. Consequently, the isolation strategy must be meticulously selected to achieve high purity and minimize co-isolated contaminants that could confound downstream functional assays, such as immune modulation studies or proteomic profiling [21].

Contemporary research employs a diverse array of isolation techniques, each presenting distinct advantages regarding yield, purity, cost and scalability. Differential ultracentrifugation remains the most widely utilized method based on particle size and density, yet it is often limited by the co-sedimentation of nonvesicular protein complexes. To overcome such limitations, researchers increasingly adopt more sophisticated approaches, including density gradient fractionation, size-exclusion chromatography and polymer-based precipitation or highly specific immunoaffinity capture targeting surface markers. The selection of an appropriate method is not merely technical but determines the validity of the biological conclusions drawn, as different techniques may isolate distinct vesicular subpopulations with varying functional cargos. Therefore, the choice of isolation protocol must be carefully aligned with the specific experimental objectives, whether aimed at high-throughput diagnostic screening or the detailed mechanistic dissection of host–pathogen interactions.

**Figure 2:**
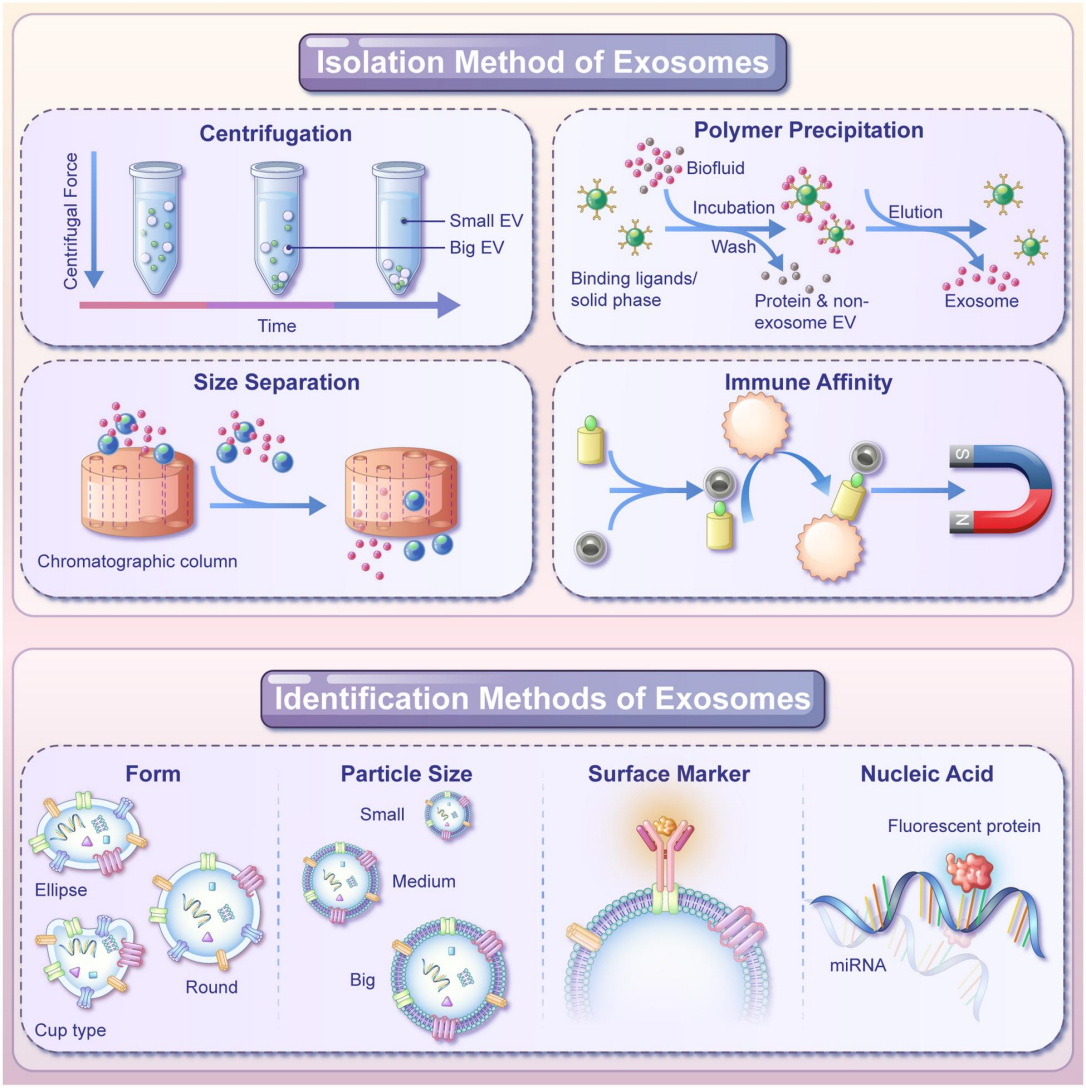
Representative isolation and identification methodologies of exosomes. The schematic outlines the standard workflows for exosomes, featuring isolation techniques (centrifugal, polymer precipitation, size-based separation and immunoaffinity capture) paired with essential characterization methods (morphological, size distribution, surface marker and nucleic acid profiling).

Following isolation, the validation of exosomal identity necessitates a comprehensive, multiparametric approach to ensure adherence to established community guidelines. This validation process typically integrates electron microscopy to visualize vesicular morphology and cup-shaped structures, nanoparticle tracking analysis (NTA) to assess size distribution and concentration and western blotting to confirm the presence of specific endosome-associated markers such as tetraspanins or Alix while demonstrating the absence of cellular contaminants (Figure 2) [22]. In bacterial infection research, it is further imperative to verify that observed effects are attributable to exosomes rather than contaminating bacterial toxins or cell wall components like lipopolysaccharide. Ultimately, the fidelity of these isolation and identification protocols profoundly influences the reliability of subsequent therapeutic assessments and our understanding of how exosomes orchestrate the complex interplay between bacterial pathogens and host immunity as depicted.

### Isolation methods of exosomes

#### Centrifugal method

Differential ultracentrifugation remains the foundational and most extensively validated methodology for exosome isolation, historically serving as the gold standard against which other techniques are benchmarked. This approach operates on the physical principle of sedimentation, where particles are fractionated based on their size and density under sequentially increasing centrifugal forces [23]. The protocol typically necessitates a stepwise escalation of force. Initial low-speed spins at approximately 300–500 *g* remove gross cellular debris and apoptotic bodies, followed by intermediate centrifugation at 10 000–20 000 *g* to eliminate larger microvesicles and organelles. The process culminates in a high-speed ultracentrifugation step exceeding 100 000 *g*, which successfully pellets the nanoscale exosomes.

Despite its conceptual simplicity and broad adoption, this method presents significant limitations in the context of clinical infectious disease research. The comprehensive separation process is notably time-intensive, often exceeding 18 h [24]. More critically, the purity of the final isolate is frequently compromised by the co-sedimentation of protein aggregates, viral particles and lipoproteins that share similar sedimentation coefficients with exosomes. In the context of bacterial infection studies, this physical overlap presents a profound challenge: bacterial outer membrane vesicles (OMVs, typically 20–250 nm) and high-molecular-weight secreted toxins frequently co-pellet with host-derived exosomes [25]. This issue is particularly acute when processing viscous, protein-rich biological fluids such as sputum from pneumonia patients or purulent exudates from abscesses, where nonvesicular contaminants and bacterial debris can mechanically entrap host exosomes, severely confounding downstream functional analyses [26].

To overcome these severe matrix effects during centrifugal isolation, specialized pretreatments must be integrated. For highly viscoelastic sputum samples, chemical mucolysis using reducing agents such as N-acetyl-L-cysteine (NALC) or low concentrations of dithiothreitol (DTT) combined with DNase I is mandatory prior to centrifugation; this step cleaves disulfide bonds within the mucin network and digests free genomic DNA, liberating mechanically trapped vesicles [27]. For pus samples heavily contaminated with necrotic tissue and intact bacteria, raw exudates must be diluted 5- to 10-fold in ice-cold phosphate-buffered saline (PBS) and subjected to an expanded, multistage serial differential pre-clearing regimen (e.g. 500 *g* for 15 min, 3000 *g* for 20 min and 12 000 *g* for 45 min) to exhaustively sediment host cellular debris and intact pathogens before high-speed pelleting [28].

To address the purity constraints inherent to simple pelleting, density gradient centrifugation was developed as a sophisticated refinement. This technique involves layering the pre-cleared sample atop a discontinuous or continuous gradient medium, such as sucrose or iodixanol [21]. During prolonged ultracentrifugation, exosomes migrate to their specific isopycnic point, typically between 1.13 and 1.19 g/mL, effectively separating them from contaminants with different buoyant densities [29]. While this method achieves superior resolution and effectively purges protein aggregates, it imposes a substantial operational burden. The requirement for meticulous gradient preparation, extended run times and subsequent labor-intensive fraction collection renders it impractical for high-throughput clinical diagnostics. Furthermore, the necessary post-isolation removal of gradient media via dialysis introduces additional risks of vesicle loss or structural damage. Consequently, density gradient centrifugation is largely reserved for mechanistic studies where establishing a pristine, contaminant-free exosome population—separated from the bulk of free bacterial factors—is paramount over yield or speed.

#### Polymer precipitation method

In response to the logistical and temporal demands of ultracentrifugation, polymer-based precipitation has emerged as a high-yield alternative favored in clinical settings where sample volume is limited or throughput requirements are high. These protocols leverage the physicochemical principle of volume exclusion. Hydrophilic polymers, most notably polyethylene glycol with molecular weights ranging from 6000 to 8000 Da, are introduced into biofluids such as serum or cell culture supernatants. By sequestering water molecules, the polymer effectively reduces the solubility of exosomes, forcing them out of the solution [26]. This dehydration process allows for the recovery of vesicular complexes via low-speed centrifugation, thereby circumventing the need for specialized ultracentrifugation equipment.

However, the mechanism is inherently nonspecific. The polymer indiscriminately precipitates other macromolecules, leading to massive contamination with immune complexes, lipoproteins, host extra-vesicular proteins, and crucially, bacterial OMVs and free toxins. This lack of purity restricts the utility of crude PEG precipitation for detailed proteomic analysis or mechanistic studies of infection, where differentiating host responses from bacterial contaminants is critical. To mitigate this, combinatorial strategies are frequently employed, such as using PEG for initial volume reduction followed by size-exclusion chromatography or density gradient steps to refine the isolate purity [30].

This optimization is particularly vital when dealing with peripheral blood (serum or plasma), where overwhelming backgrounds of soluble albumin and lipoproteins (chylomicrons, VLDL, LDL and HDL) lead to catastrophic co-precipitation during PEG processing [31]. To optimize polymer precipitation for clinical blood samples, plasma collected with anticoagulants such as EDTA or acid citrate dextrose-A (ACD-A) is strongly preferred over serum to prevent platelet activation during clotting, which artificially inflates vesicle counts. Furthermore, the subsequent integration of lipoprotein-depletion columns (e.g. targeting apolipoprotein B) is highly recommended to refine the clinical blood isolate [32].

A distinct variation within precipitation methodologies is lectin-based affinity precipitation, which transitions from volume exclusion to specific biochemical recognition. This approach exploits the distinct glycan profiles present on exosome surfaces by using lectins like concanavalin A or wheat germ agglutinin to bind vesicular glycoconjugates [33]. While this method offers enhanced specificity compared to PEG by targeting surface features, it traditionally suffers from long incubation times and potential nonspecific binding. Recent advancements have integrated solid-phase technologies, immobilizing lectins onto magnetic beads or chromatographic columns. This modification allows for rapid binding and efficient elution with competitive sugars, significantly streamlining the workflow. Ultimately, the selection between PEG and lectin-based methods relies on the experimental priority, balancing the need for massive yields against the requirement for subpopulation specificity.

#### Size-based separation method

Size-based separation techniques exploit the defined nanoscale dimensions of exosomes to distinguish them from the complex milieu of biological fluids. Two primary methodologies, ultrafiltration and size-exclusion chromatography (SEC), dominate this category and offer distinct advantages for preserving vesicle biological activity.

Ultrafiltration utilizes semipermeable membranes with specific molecular weight cutoffs, typically 100 kDa or 300 kDa, to physically retain exosomes while allowing smaller soluble proteins and metabolites to pass through [34]. This method is notably faster than ultracentrifugation and does not require expensive instrumentation. However, the application of pressure or centrifugal force can induce shear stress, potentially leading to vesicle deformation or rupture. Furthermore, in clinical sputum samples, ultrafiltration membranes suffer from catastrophic clogging by polymeric mucins unless the matrix is chemically liquefied and passed through a dual-stage serial filtration (first a 0.45 μm filter to catch mucus clumps, then a 0.22 μm filter) [32]. Importantly, while ultrafiltration can remove small, free bacterial toxins, it fails to separate host exosomes from larger multiprotein toxin complexes or bacterial OMVs due to the lack of size discrimination at the nanoscale [35].

SEC offers a gentler, nonshearing separation mechanism relying on gravity-flow or low-pressure systems. As the sample traverses a column packed with porous stationary-phase beads, smaller molecules—including abundant host soluble proteins (such as albumin and immunoglobulins) and free bacterial toxins—penetrate the pores and experience a longer path length, delaying their elution [36]. Larger particles, including host exosomes, are excluded from these pores and elute earlier in the void volume, typically using phosphate-buffered saline as an eluent. This preservation of structural integrity and native surface protein conformation is exceptionally vital for functional assays in bacterial infection research, where studying exosome-mediated immune modulation or virulence factor transfer is intended [37].

Nevertheless, challenges persist. SEC requires significant sample dilution, potentially necessitating a post-isolation concentration step. More importantly, SEC struggles to differentiate host-derived exosomes from bacterial OMVs, as both vesicle types possess overlapping hydrodynamic radii and elute simultaneously in the void volume. Additionally, achieving high-resolution separation demands precise column calibration, and column reuse carries risks of cross-contamination. Notably, recent technological advancements, exemplified by commercial kits (e.g. qEV kits from iZON Science), have substantially streamlined workflows, reducing separation times to approximately 15 min while retaining the core gentle separation principle [38].

An emerging advanced technique in this domain is field-flow fractionation (FFFF). Unlike the passive separation of SEC, FFFF actively sorts particles within a thin channel by applying a cross-flow field perpendicular to the main laminar flow. Larger particles are driven toward the accumulation wall and elute slower, while smaller particles remain in the faster central stream [39]. This method offers high-resolution separation of exosome subpopulations with minimal perturbation. However, the high complexity and cost of the instrumentation currently limit its widespread adoption outside of specialized physical chemistry laboratories [40].

#### Immunoaffinity capture method

Immunoaffinity capture represents the pinnacle of isolation specificity, leveraging the molecular recognition of surface antigens to selectively retrieve exosomes. This method immobilizes antibodies targeting constitutive exosomal markers, such as the tetraspanins CD63, CD81 and CD9, onto solid matrices like magnetic beads or microfluidic chips. The technique excels in producing highly pure isolates by specifically binding vesicles expressing the target antigen while washing away nonspecific contaminants [41].

In the context of clinical infectious disease research, immunoaffinity capture offers an unparalleled advantage: it serves as the most reliable strategy to rigorously separate host-derived exosomes from bacterial OMVs and extracellular toxins. Because OMVs lack mammalian tetraspanins and toxins lack specific host surface markers, targeting host-specific antigens allows for the pristine enrichment of host vesicles [42]. Moreover, in highly chaotic clinical matrices like purulent exudates (pus), immunoaffinity capture stands out as the ultimate refinement tool capable of pulling intact host exosomes directly out of a background of neutrophil extracellular traps (NETs) and lysed bacterial components. This high purity is indispensable for unraveling complex host–pathogen interactions, such as characterizing host exosomes derived specifically from *Mycobacterium tuberculosis*-infected macrophages without interference from bacterial cargo [43].

However, the reliance on surface markers introduces a critical vulnerability in the context of infectious diseases. Bacterial infections are dynamic processes that can significantly alter the host cell surface proteome, potentially downregulating the very markers used for capture. Consequently, the reliance on a single marker may lead to the biased selection of subpopulations, failing to capture the full spectrum of infection-associated vesicles [44]. Additionally, clinical optimization for pus samples requires incubating the diluted matrix with an enzymatic cocktail (combining DNase I and collagenase) to dissolve the sticky NET structures, alongside the mandatory addition of potent protease and phosphatase inhibitors. This step is vital to protect the target exosomal surface epitopes from degradation by the abundant endogenous lysosomal enzymes found in purulent environments, thereby ensuring efficient antibody–antigen binding [45]. Furthermore, the strong antibody–antigen interaction poses challenges for eluting intact, functional vesicles without using harsh buffers that might compromise biological activity. To address these challenges, optimization strategies now emphasize the use of combinatorial antibody panels to broaden capture efficiency and the development of gentle, competitive elution protocols. While the yield is generally lower and the costs higher compared to other methods, immunoaffinity capture remains the method of choice when specificity and purity are the absolute priorities [46] (Table 1).

**Table 1:** Comparison of common exosome isolation methods, their limitations in infection studies and optimal application scenarios.

Isolation method	Purity	Yield	Time	Cost	Limitations & challenges in infection studies	Optimal application scenarios
Differential ultracentrifugation (UC)	Low to moderate	High	Long (>12 h)	Moderate (equipment)	Fails to distinguish host exosomes from bacterial OMVs and high-MW toxins; they frequently co-pellet due to similar sedimentation coefficients.	Pre-cleared large-volume samples; suitable for initial bullet-enrichment of cell culture supernatants or diluted biofluids where high initial yield is prioritized [47].
Density gradient centrifugation	High	Low	Very long (>18 h)	Moderate	Effectively removes free toxins and protein aggregates, but bacterial OMVs sharing similar buoyant densities (1.13–1.19 g/mL) may still co-isolate.	In-depth mechanistic studies; defining pristine host exosome cargo profiles (proteomics/RNA-seq) where contaminant-free background is paramount over speed [48].
Polymer precipitation (e.g. PEG)	Low	Very high	Short (1–2 h)	Low	Indiscriminately co-precipitates host exosomes, bacterial OMVs and free extracellular toxins, leading to severe background contamination.	High-throughput clinical screening; initial volume reduction of small-volume blood or urine samples, usually combined with downstream SEC or density gradient steps [49].
Size-exclusion chromatography (SEC)	Moderate to high	Moderate	Short (∼0.5 h)	Moderate	Preserves vesicle integrity and eliminates small soluble toxins, but fails to separate host exosomes from OMVs due to overlapping size distributions (30–150 nm).	Functional and behavioral assays; processing pretreated/mucolysed sputum or blood samples where maintaining the native conformation of surface proteins is vital [37].
Immunoaffinity capture	Very high	Low	Moderate (2–12 h)	High	Gold standard for infection studies; precisely separates host vesicles from OMVs/toxins by targeting host markers (CD63/CD81). However, suffers from low yield and high cost.	Chaotic clinical matrices (e.g. Pus); isolating specific host-derived vesicle subpopulations from heavy bacterial backgrounds or distinguishing infected from uninfected host vesicles [50].

### Identification methods of exosomes

#### Morphological characterization

Transmission electron microscopy (TEM) and SEM are widely regarded as gold-standard methods for visualizing the characteristic morphology of exosomes, providing definitive confirmation of their identity. In conventional TEM analysis, exosome samples are adsorbed onto a coated grid, negatively stained with agents such as uranyl acetate or phosphotungstic acid and imaged under high vacuum. This reveals the classic exosomal profile: cup-shaped vesicles bounded by a distinct lipid bilayer, with diameters typically ranging from 30 to 150 nm [51]. For example, exosomes isolated from the serum of *Cynoglossus semilaevis* infected with *Vibrio harveyi* or from supernatants of *M. tuberculosis*-infected macrophages have been verified by TEM to display this signature morphology, supporting the successful isolation of intact vesicles [52].

SEM provides complementary high-resolution surface topological data, although its requisite sample dehydration and conductive coating may introduce artifacts. Critically, the visualization of a bilayered membrane structure via electron microscopy transcends mere imaging; it serves as a fundamental criterion validating that the isolated entities are indeed intact, membrane-bound extracellular vesicles consistent with the definition of exosomes and not mere protein aggregates or cellular debris [53]. This morphological validation is especially important when studying exosomes in the context of bacterial infection, where pathogens such as *Glaesserella parasuis*, *S. aureus* or *M. tuberculosis* have been demonstrated to modulate exosome biogenesis and release [54, 55]. However, electron microscopy remains low-throughput, requires specialized expertise and equipment and may distort native vesicle structures because of fixation, staining or dehydration.

The need to examine exosomes in a near-native state has promoted the use of cryo-electron microscopy (cryo-EM). By rapidly vitrifying samples to preserve their native hydrated structure, cryo-EM effectively circumvents the artifacts associated with conventional chemical fixation and dehydration. When integrated with single-particle analysis, this technique enables high-resolution three-dimensional reconstruction, revealing accurate vesicle dimensions and even capturing dynamic processes such as membrane fusion with high fidelity [56]. Its routine application, however, is limited by high instrumentation costs and computationally intensive data processing.

Atomic force microscopy (AFM) provides complementary three-dimensional topographic and nanomechanical information. By physically probing vesicle surfaces, AFM can measure height profiles and mechanical properties such as Young’s modulus without requiring fixation [56]. This capability is especially useful for assessing how bacterial toxins or infection-induced changes affect exosome membrane stiffness and integrity. Overall, the field is moving toward multimodal strategies that combine SEM, cryo-EM, TEM and AFM to capture both population-level heterogeneity and individual vesicle integrity.

#### Size distribution analysis

Complementary to morphological validation, the precise determination of particle size distribution is indispensable for confirming that the isolate falls within the canonical exosome diameter range of 30–150 nm [57]. This quantitative assessment is dominated by two principal technologies, namely dynamic light scattering (DLS) and NTA. DLS operates by analyzing the fluctuations in scattered light intensity caused by the Brownian motion of particles in suspension. While DLS provides a rapid and statistically robust estimation of the average hydrodynamic diameter and polydispersity index of the entire population, it is inherently biased toward larger particles which scatter light more intensely. This limitation renders DLS less effective for resolving polydisperse samples typical of biological fluids, where larger contaminants can mask the signal of smaller exosomes [58].

NTA overcomes the ensemble averaging limitations of DLS by utilizing a laser-illuminated microscopic system to visualize and track the Brownian motion of individual particles. By calculating the mean squared displacement of each track, NTA derives the diffusion coefficient and subsequent hydrodynamic diameter for every particle, generating a high-resolution concentration and size distribution profile [59]. This single-particle approach provides a more accurate representation of polydisperse samples and can distinguish exosomes from larger vesicles or smaller contaminants more effectively than DLS [60]. In the context of bacterial infections, NTA is particularly advantageous as it allows researchers to quantify subtle shifts in vesicle size profiles that may occur in response to pathogen exposure. For instance, infection with intracellular bacteria often stimulates the release of distinct vesicle subpopulations or alters the balance between exosomes and larger microvesicles. However, a persistent challenge in infection biology is the size overlap between host-derived exosomes and bacterial OMVs. Standard NTA cannot distinguish between these two populations based on size alone.

To address the resolution limits of NTA regarding refractive index and size discrimination, single-particle interferometric reflectance imaging sensing (SP-IRIS) has been developed as a next-generation solution. Unlike scattering-based methods, SP-IRIS relies on the interference of light reflected from the sensor surface and the particle, providing superior sensitivity for detecting smaller vesicles down to 40 nm. Comparative studies have demonstrated that SP-IRIS can resolve mixed populations of 40 nm and 70 nm particles that NTA would aggregate into a single peak [61]. Furthermore, SP-IRIS systems often integrate fluorescence capabilities, enabling the phenotyping of individual vesicles based on surface markers. This allows for the specific discrimination of host exosomes from bacterial vesicles within a mixed sample, a capability that represents a significant leap forward for characterizing the complex extracellular milieu of infected tissues [62].

#### Surface marker identification

Following biophysical characterization, the biochemical validation of specific surface markers is required to definitively classify isolated vesicles as exosomes and assess their purity. The International Society for Extracellular Vesicles (ISEV) guidelines recommend a combinatorial approach that demonstrates the presence of transmembrane proteins enriched in exosomes, such as the tetraspanins CD9, CD63 and CD81, as well as cytosolic proteins involved in exosome biogenesis like ALIX and TSG101. Western blotting serves as the primary method for this validation, providing semiquantitative data on the enrichment of these positive markers while crucially verifying the absence of negative markers. Negative markers are proteins exclusively associated with intracellular compartments such as the endoplasmic reticulum (ER; e.g. Calnexin) or mitochondria (e.g. cytochrome C). Their absence indicates limited contamination by cellular debris or organelles [63].

In the specific landscape of bacterial infections, surface marker analysis assumes a dual role. Beyond mere identification, it serves as a tool for probing the pathological state of the host. Bacterial pathogens can dramatically alter the protein composition of host exosomes, leading to the downregulation of conventional markers or the incorporation of infection-specific molecules. For example, proteomic analyses validated by Western blotting have shown that stimulation of epithelial cells with bacterial components can upregulate specific cargo proteins involved in immune signaling. Moreover, rigorous surface profiling is the most reliable method to distinguish host exosomes from co-isolated bacterial OMVs, which carry distinct bacterial outer membrane proteins but lack mammalian tetraspanins. Consequently, advanced flow cytometry techniques, including nano-flow cytometry capable of resolving single vesicles, are increasingly employed to analyze the heterogeneity of surface marker expression, allowing researchers to pinpoint specific exosome subpopulations that act as mediators of the host immune response during infection.

#### Nucleic acid profiling

The analysis of exosomal cargo extends beyond proteins to include a diverse array of nucleic acids, specifically microRNAs (miRNAs), messenger RNAs (mRNAs) and circular RNAs (circRNAs). These molecules are not merely passive passengers but functional effectors that facilitate genetic communication between the infected cell and the host immune system. The profiling of this RNA cargo typically begins with extraction using phenol-chloroform-based methods or specialized kits, followed by analysis via quantitative real-time PCR (qRT-PCR) or high-throughput RNA sequencing (RNA-seq).

Targeted qRT-PCR is invaluable for validating specific hypotheses regarding known immune-regulatory RNAs. For instance, studies on *V. harveyi* infection demonstrated through qRT-PCR that specific miRNAs, such as miR-133-3p, are significantly upregulated in serum exosomes and actively promote the expression of proinflammatory cytokines like TNF-α and IL-6 in recipient cells [52]. Conversely, high-throughput sequencing provides an unbiased, comprehensive view of the exosomal transcriptome, enabling the discovery of novel biomarkers and regulatory networks without prior knowledge of the target. This approach is essential for mapping the global landscape of host–pathogen interactions, as it can reveal complex changes in RNA profiles, such as the differential expression of circRNAs in *G. parasuis* infection, which may mediate host resistance mechanisms [64]. Integrating nucleic acid profiling with proteomic and biophysical analyses can therefore provide a more comprehensive understanding of how exosomes regulate bacterial pathogenesis, immune activation and tissue damage.

For *in vivo* antibacterial applications, strict quality control and sterility assessment are essential to avoid confounding effects and safety risks caused by bacterial OMVs, toxins, LPS, viral particles or protein aggregates. Exosome preparations should be produced under sterile and mycoplasma-free conditions, preferably using exosome-depleted or chemically defined media. Isolation protocols should combine differential centrifugation with orthogonal purification methods, such as density-gradient ultracentrifugation, size-exclusion chromatography, ultrafiltration or tangential-flow filtration, to remove bacterial vesicles, protein aggregates and other non-exosomal contaminants. Each batch should be characterized by nanoparticle tracking analysis, transmission electron microscopy and exosomal marker analysis, together with sterility testing, mycoplasma testing, endotoxin/LPS quantification, bacterial and fungal culture and viral safety assessment. Potential bacterial OMV contamination can be evaluated by LPS assays, bacterial DNA detection, and bacterial outer membrane protein markers, whereas protein aggregates can be monitored by particle-to-protein ratio, size-exclusion chromatography, density-gradient purification, TEM morphology and protease-sensitivity analysis. Therefore, release criteria should include sterility, mycoplasma negativity, low endotoxin levels, absence of bacterial OMV markers, defined particle size distribution, acceptable particle/protein ratio and batch-to-batch reproducibility before *in vivo* administration.

## Classification of exosomes

**Figure 3:**
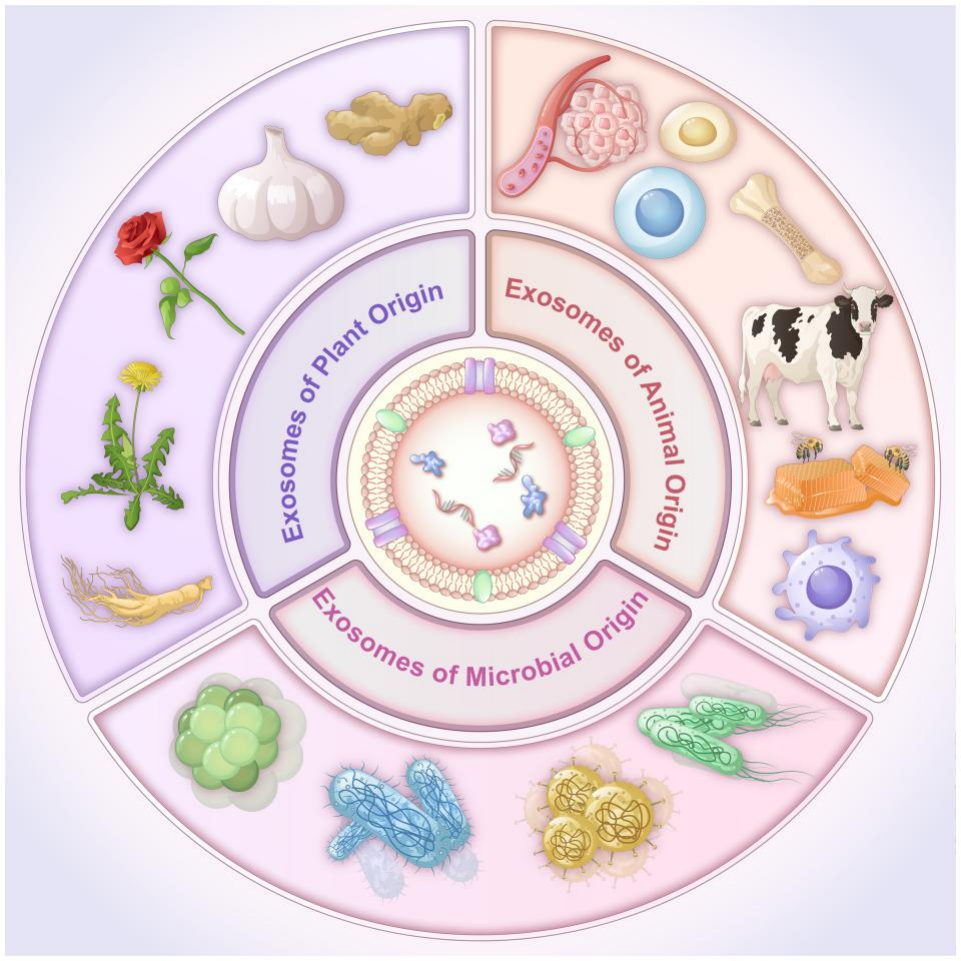
Classification of exosomes: plant-derived exosomes (PDEs), animal-derived exosomes, and microbial-derived exosomes.

Exosomes represent a phylogenetically conserved mechanism of intercellular communication that transcends the boundaries of the animal kingdom to encompass plants and microbes. While these vesicles share fundamental biophysical properties such as nanoscale dimensions and lipid bilayer structures, their biological origin dictates profound differences in molecular composition, biogenesis pathways and functional capabilities. A comparative analysis of exosomes derived from distinct biological kingdoms reveals a spectrum of unique attributes. Animal-derived exosomes are integral to complex immune system regulation, whereas plant-derived nanovesicles often function as mediators of cross-kingdom interactions, and microbial vesicles serve as critical virulence factors or defense mechanisms. Understanding these origin-dependent nuances is not merely taxonomical but essential for translational medicine. It allows researchers to exploit the specific evolutionary advantages of each class, such as the intrinsic biocompatibility of plant vesicles or the immunogenic potential of bacterial vesicles, to engineer precise diagnostic and therapeutic tools against infectious diseases, as illustrated (Figure 3).

### Plant-derived exosomes

PDEs, frequently referred to in the literature as ELNs, are an emerging therapeutic platform isolated from a wide range of edible and medicinal plants [65]. Although their biogenesis shares similarities with mammalian multivesicular body pathways, PDEs possess a distinct structural and biochemical identity. They are naturally encapsulated in a lipid bilayer enriched with plant-specific lipids such as phosphatidic acid and galactolipids, which contribute to their unique stability profiles. This is particularly evident in PDEs isolated from traditional Chinese botanicals such as *Zingiber officinale* (ginger), *Panax ginseng* and *Rhodiola rosea*, where vesicles can act as natural carriers of plant-derived bioactive compounds. This intrinsic molecular composition underpins their potent biological activities and positions them as promising candidates for managing bacterial infections through mechanisms that are distinct from mammalian vesicles [66].

The therapeutic potential of PDEs in the context of infectious disease is defined by a dual mechanism of action encompassing direct antimicrobial activity and host immunomodulation. Research indicates that PDEs can exert bacteriostatic or bactericidal effects through the direct disruption of bacterial cell walls or by interfering with quorum-sensing systems essential for biofilm formation and virulence factor secretion [67]. For instance, vesicles derived from ginger have demonstrated dose-dependent antibacterial activity against periodontal pathogens like *Porphyromonas gingivalis*, effectively reducing bacterial load [68]. Complementing this direct attack, PDEs function as sophisticated immunomodulators capable of mitigating the excessive inflammation that often complicates severe bacterial infections. They can reprogram host immune responses, shifting macrophage polarization from a pro-inflammatory M1 phenotype to a restorative M2 state and suppressing the release of damaging cytokines. Specific examples include ginseng-derived vesicles which deliver anti-inflammatory ginsenosides (Rg3, Rb1 and Rk) to suppress NF-κB signaling pathways and *Rhodiola* or dandelion-derived vesicles which have been shown to inhibit pulmonary fibrosis and modulate T-cell differentiation [69]. Thus, PDEs may provide a dual benefit by targeting pathogens while limiting infection-associated immunopathology.

Beyond their bioactive profiles, the clinical translation of PDEs is supported by favorable pharmacological properties, including safety, scalability and delivery efficiency. As products of dietary sources, PDEs generally exhibit excellent biocompatibility and a low risk of immunogenicity or zoonotic pathogen transmission compared to mammalian cell-derived counterparts. A defining advantage of PDEs is their remarkable structural stability, particularly their resistance to degradation by gastric acids and proteolytic enzymes in the gastrointestinal tract. This robustness facilitates oral administration, a highly desirable route that improves patient compliance while enabling the delivery of labile therapeutic cargo to the gut mucosa or systemic circulation [70]. Furthermore, evidence suggests that PDEs possess inherent tissue-homing properties determined by their specific lipid-protein surface composition. Recent breakthroughs have even demonstrated that PDEs from sources like *Rosa rugosa* can cross the blood-brain barrier to deliver therapeutics to the central nervous system without the need for chemical modification [71]. Collectively, the integration of inherent antimicrobial potency, low toxicity and oral bioavailability highlights PDEs as promising candidates for next-generation therapies against difficult-to-treat bacterial infections.

### Animal-derived exosomes

Animal-derived exosomes represent a physiologically heterogeneous and functionally sophisticated class of extracellular vesicles that serve as ubiquitous mediators of intercellular communication within the metazoan environment. Distributed across all biological fluids, including blood plasma, saliva, urine, milk and tissue interstitial spaces, these lipid bilayer-enclosed nanovesicles function as stable carriers for a diverse bioactive cargo of proteins, lipids and nucleic acids [72]. Unlike their plant or microbial counterparts, animal-derived exosomes act as integral components of the host neuro-immuno-endocrine network, orchestrating complex systemic responses to maintain homeostasis. In the specific landscape of bacterial pathogenesis, these vesicles transcend simple messenger functions to become active participants in the host–pathogen evolutionary arms race. They facilitate a dynamic molecular dialogue that governs innate immune activation, adaptive memory formation, tissue regeneration following infectious injury and the resolution of inflammation. Consequently, dissecting the functional heterogeneity of exosomes based on their specific cellular origin is paramount for understanding host defense mechanisms and for harnessing these biological nanoparticles as targeted therapeutic agents or precise diagnostic biomarkers.

#### Exosomes from immune cells

The immune system utilizes exosomes as rapid, distinct vectors to coordinate the multilayered host response against bacterial invasion. These vesicles, secreted by both innate and adaptive immune cells, function as pivotal orchestrators that bridge pathogen recognition with effector functions.

Exosomes derived from innate immune cells, such as natural killer (NK) cells and macrophages, serve as the first line of defense. NK cell-derived exosomes are armed with cytotoxic payloads including perforin and granzymes, enabling them to exert direct bactericidal effects or induce apoptosis in infected host cells to eliminate intracellular niches of pathogens like *M. tuberculosis* [73].

Concurrently, macrophage-derived exosomes function as critical signal amplifiers. Upon encountering bacterial stimuli, these vesicles are enriched with pathogen-associated molecular patterns and proinflammatory cytokines. They transfer these danger signals to naïve immune cells, thereby activating NF-κB signaling pathways and catalyzing the production of TNF-α and IL-6 to potentiate bacterial clearance [74, 75]. However, this communication channel is vulnerable to pathogen subversion. Certain bacteria, such as *G. parasuis*, can actively manipulate host machinery to downregulate specific exosomal cargos like circHIF1α, effectively dampening DNA damage responses and facilitating immune evasion [54].

On the adaptive immunity front, exosomes from T and B lymphocytes modulate the intensity and duration of the immune response. While exosomes from exhausted CD8+ T cells in chronic infections may propagate immune dysfunction, B cell-derived exosomes have emerged as potent regulators of inflammation. Engineered B cell exosomes carrying specific miRNAs, such as miR-155, have demonstrated the capacity to suppress lipopolysaccharide-induced signaling, highlighting their potential to prevent the “cytokine storm” often associated with septic shock [76]. Thus, immune-cell-derived exosomes can both promote pathogen elimination and help restrain tissue-damaging inflammation.

#### Exosomes from stem cells

In the aftermath of bacterial infection, the restoration of tissue integrity is as critical as pathogen clearance. Exosomes derived from stem cells, particularly mesenchymal stem cells (MSCs), act as potent paracrine mediators of regeneration and immunomodulation. Their therapeutic utility stems from their ability to recapitulate the beneficial effects of stem cell transplantation while circumventing the risks of teratoma formation and immune rejection associated with whole-cell therapies.

MSC-derived exosomes function through a dual mechanism of inflammation suppression and tissue repair promotion. For example, exosomes isolated from adipose-derived MSCs facilitate the healing of infected diabetic wounds by simultaneously activating PI3K/AKT and MAPK/ERK pro-survival pathways. This molecular signaling accelerates angiogenesis and re-epithelialization while concurrently suppressing neutrophil infiltration to prevent chronic inflammation. Similarly, exosomes from human umbilical cord MSCs have shown remarkable efficacy in sepsis models. They effectively reprogram macrophages from a proinflammatory M1 phenotype to an anti-inflammatory M2 state, thereby reducing systemic levels of IL-1β and IL-6 while enhancing the production of the regulatory cytokine IL-10 [77]. This immunomodulatory capability is critical for preventing multiorgan failure during severe bacteremia. Further expanding their therapeutic scope, exosomes from endothelial progenitor cells contribute to vascular protection by inhibiting endothelial-mesenchymal transition and reducing vascular leakage, key pathophysiological features of sepsis-induced cardiovascular dysfunction [78].

Additionally, bioengineering strategies have enhanced the clinical applicability of these vesicles. The encapsulation of bone marrow MSC exosomes within biomaterial scaffolds, such as chitosan hydrogels, prolongs their retention at infection sites, creating a sustained-release system that synergistically combats inflammation and accelerates wound closure in recalcitrant ulcers [79].

#### Exosomes from specialized somatic cells

Beyond professional immune and stem cells, specialized somatic cells release exosomes that function as local defenders, directly modulating the microenvironment at the site of infection. These tissue-specific vesicles often employ sophisticated molecular strategies to counteract bacterial virulence factors.

A striking example of this inter-kingdom interference is observed in airway epithelial cells. These cells secrete exosomes containing specific miRNAs, such as let-7b-5p, which are taken up by pathogens like *P. aeruginosa*. Remarkably, these host miRNAs can target and downregulate bacterial quorum-sensing genes, effectively disrupting biofilm architecture and rendering the bacteria susceptible to β-lactam antibiotics [80]. This finding suggests that host vesicles can directly interfere with bacterial defense programs.

In the circulatory system, erythrocyte-derived exosomes released during systemic infection act as mobile scavengers. They transport broad-spectrum antimicrobial proteins, including lactoferrin and interferon-induced transmembrane proteins, which sequester essential nutrients like iron or directly disrupt bacterial membranes [81]. Meanwhile, in the central nervous system, neuronal exosomes can shuttle pathogenic miRNAs to activate microglia, highlighting a mechanism by which local infections can trigger neuroinflammatory responses. These findings illustrate that somatic cell exosomes are not passive bystanders but active agents in mucosal and systemic barrier immunity.

#### Exosomes from animal noncellular sources

Exosomes isolated directly from animal biofluids represent a unique category that serves dual roles as accessible diagnostic reservoirs and intrinsic therapeutic agents. Because these vesicles originate from a multitude of cell types *in vivo*, they provide a comprehensive molecular snapshot of the host’s physiological state.

Diagnostically, plasma exosomes offer a real-time liquid biopsy window into infection progression. Specific alterations in their RNA cargo, such as elevated miR-150 and suppressed let-7b levels, have been identified as reliable biomarkers for predicting organ dysfunction and clinical outcomes in patients with bacteremia and sepsis [82]. These signatures enable clinicians to monitor disease severity with higher sensitivity than traditional inflammatory markers.

Therapeutically, exosomes found in secretory fluids exhibit inherent antimicrobial properties shaped by evolution. Bovine colostrum exosomes, for instance, are naturally enriched with immune proteins that inhibit the growth of methicillin-resistant *S. aureus* (MRSA) by neutralizing pore-forming toxins and disrupting bacterial bioenergetics [83]. Similarly, vesicles derived from honey products deliver bee-specific AMPs that selectively target cariogenic pathogens like *Streptococcus mutans* via nanoscale membrane perturbation while preserving the commensal oral flora [84]. However, it is crucial to acknowledge that the functional impact of biofluid-derived exosomes is context-dependent. In patients with co-morbidities such as gastric cancer, tumor-derived exosomes circulating in fluids may exacerbate concurrent infections by suppressing macrophage phagocytosis and promoting neutrophil polarization toward immunosuppressive phenotypes [85]. Therefore, clinical use of biofluid-derived exosomes requires careful evaluation of disease context, vesicle origin and functional cargo.

### Microbial-derived extracellular vesicles

To maintain strict scientific accuracy, it is essential to distinguish prokaryotic and microalgal vesicles from classical mammalian exosomes. In eukaryotic organisms, “exosomes” specifically denote nano-sized vesicles (30–150 nm) generated via the endosomal multivesicular body (MVB) pathway. Conversely, prokaryotes lack an endomembrane system; thus, bacterial structures such as OMVs from Gram-negative bacteria, as well as outer-inner membrane vesicles (OIMVs), cytoplasmic membrane vesicles (CMVs) and tubular membrane structures (TSMs) from Gram-positive species and mycobacteria, arise from distinct cell envelope blebbing or cell death mechanisms. While some literature loosely utilizes terms like “microbial exosomes” due to operational similarities in size distribution and functional cargo delivery, this review employs the broader, more precise classification of “microbial-derived extracellular vesicles (microbial EVs)”—encompassing both bacterial extracellular vesicles (BEVs) and microalgal EVs (MEVs)—to respect these biogenetic differences while discussing their therapeutic potential alongside plant and animal counterparts.

Bacteria possess a sophisticated secretory capacity that extends beyond soluble proteins to include the constitutive and inducible release of diverse lipid-bilayer nanostructures collectively termed bacterial extracellular vesicles (BEVs). Ranging typically from 20 to 400 nm in diameter, these vesicles represent a fundamental mechanism of microbial interaction that is evolutionarily conserved yet structurally heterogeneous. Current ultrastructural classification distinguishes several subtypes based on their biogenesis and membrane composition, encompassing OMVs derived from Gram-negative bacteria, as well as OIMVs, CMVs and TSMs, which are increasingly recognized in Gram-positive species and mycobacteria [86]. Functioning as stable, long-circulating bioactive carriers, BEVs facilitate intricate interspecies communication and host-microbe crosstalk by transporting a concentrated and complex cargo of proteins, lipids, nucleic acids and virulence factors. Within microbial communities, these vesicles act as public goods that regulate essential population-level behaviors including the dissemination of antibiotic resistance genes, the cooperative assembly of biofilms and the modulation of nutrient acquisition. Simultaneously, in the context of infection, BEVs exert profound effects on host physiology by acting as long-range delivery vehicles for microbial effector molecules [87].

The functional duality of BEVs, serving as both potent mediators of pathogenesis and potential platforms for immunomodulation, is vividly illustrated by the contrasting roles of vesicles derived from specific pathogens. MRSA serves as a paradigm for vesicular virulence. MRSA-derived BEVs encapsulate potent virulence factors such as pore-forming toxins and superantigens within a protective lipid membrane. This packaging strategy effectively shields the cargo from extracellular proteases and neutralizing antibodies, thereby enhancing stability and facilitating targeted delivery to distant host tissues. Critically, these vesicles exploit endocytic pathways to enter host cells. Upon internalization, the vesicular cargo, particularly pore-forming toxins, disrupts endosomal membranes to facilitate endolysosome escape. This cytosolic access allows bacterial effectors to subvert host immune signaling and induce cytotoxicity, a mechanism that not only drives disease progression but also offers specific targets for novel vaccine designs and diagnostic biomarker discovery [88].

Conversely, the study of *Escherichia coli* OMVs highlights the immunomodulatory and therapeutic potential of microbial vesicles. Gram-negative bacteria like *E. coli* dynamically modulate OMV biogenesis in response to physiological stressors such as nutrient limitation or membrane stress. These stress-induced OMVs display a high density of pathogen-associated molecular patterns (PAMPs), including lipopolysaccharide and outer membrane proteins, on their surface. This molecular signature makes them potent activators of innate immune signaling pathways. Leveraging this inherent immunostimulatory capacity, researchers are increasingly exploring engineered OMVs as vaccine adjuvants and antigen delivery platforms. Their ability to efficiently load and present tumor antigens or specific bacterial epitopes to dendritic cells enables them to prime potent, antigen-specific T-cell responses, thereby bridging the gap between innate and adaptive immunity in immunotherapeutic applications [89].

Expanding the horizon of microbial-derived vesicles beyond bacteriology, extracellular vesicles from eukaryotic microalgae have emerged as a promising frontier in sustainable nanomedicine. Exemplified by vesicles derived from *Tetraselmis chuii*, microalgal EVs (MEVs) offer unique advantages over bacterial or mammalian systems. As the most abundant photoautotrophs on Earth, microalgae utilize photosynthesis to generate energy-rich molecules like ATP and NADPH, a foundational process that supports cost-effective, scalable and carbon-neutral biomanufacturing. Beyond production benefits, MEVs exhibit distinct biological properties, including high biocompatibility and low immunogenicity compared to bacterial vectors. They transfer a bioactive cargo capable of conferring specific functional traits such as osteotropism, powerful antioxidative capacity and anti-inflammatory activity [90]. Consequently, microalgal EVs represent a sustainable and versatile therapeutic vector with significant potential for treating chronic inflammatory conditions and bacterial infections where tissue regeneration is required.

## Therapeutic strategies of exosomes against bacterial infections

### Direct antibacterial activity of exosomes

The escalating global crisis of antimicrobial resistance necessitates a paradigm shift from traditional pharmacotherapy toward innovative biological strategies capable of neutralizing both planktonic pathogens and recalcitrant biofilms. In this context, exosomes have emerged as multifaceted therapeutic agents that transcend the limitations of conventional antibiotics. Unlike small-molecule drugs, which typically act on single intracellular targets, exosomes function as sophisticated, nanoscale delivery vehicles that exert antimicrobial effects through a coordinated arsenal of mechanisms. These include the direct physical disruption of bacterial envelopes, the delivery of potent AMPs and the sophisticated genetic silencing of virulence or resistance factors [91]. This intrinsic multifunctionality positions exosomes as a promising next-generation modality with a reduced propensity for inducing resistance.

#### Physical disruption and antimicrobial peptide delivery

**Figure 4:**
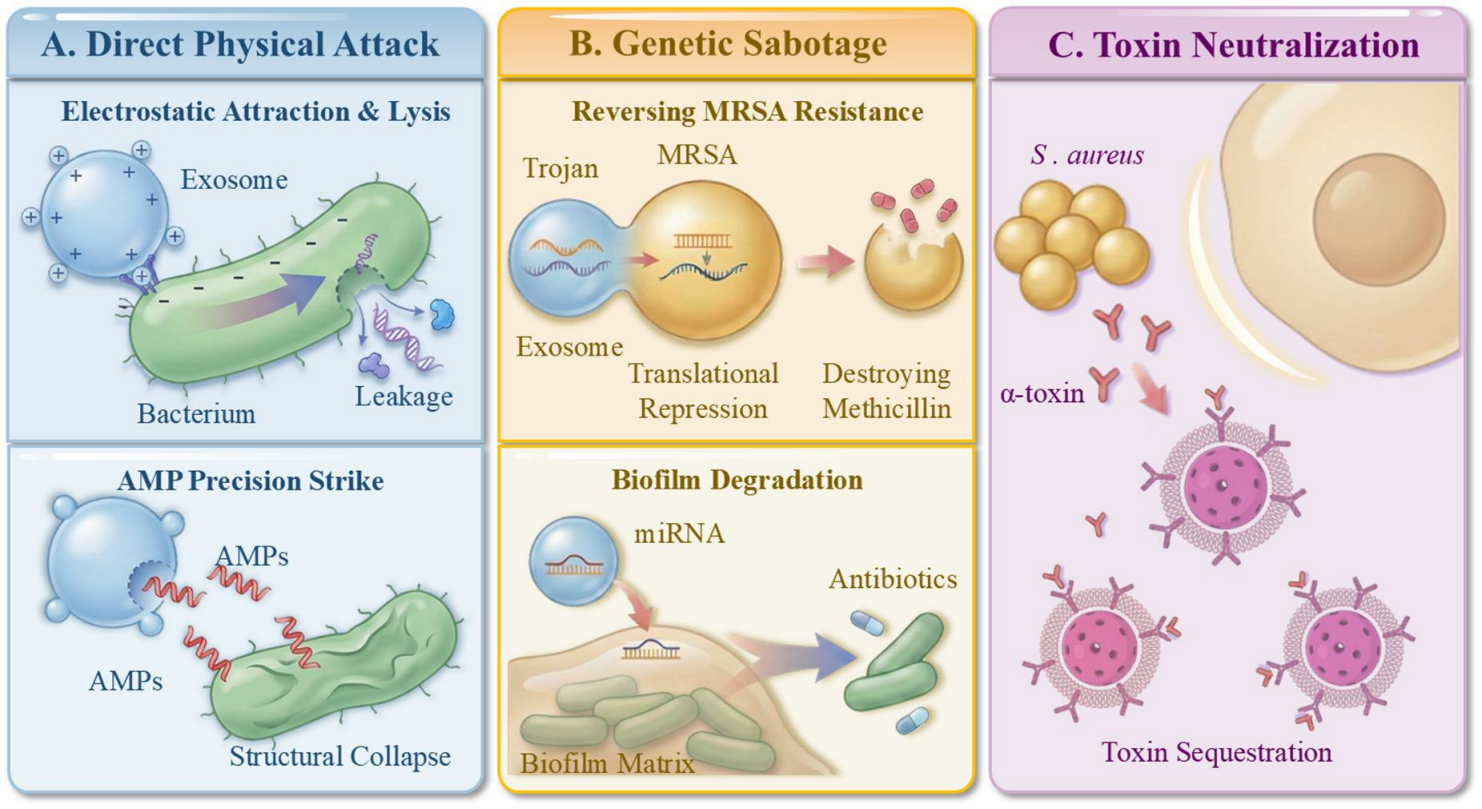
This schematic illustrates three targeted antibacterial mechanisms of therapeutic vesicles: (**A**) direct physical attack via electrostatic lysis and antimicrobial peptide (AMP) precision strikes; (**B**) genetic sabotage using specific nucleic acids to reverse MRSA resistance and degrade biofilms; and (**C**) toxin neutralization by deploying decoy receptors to sequester bacterial toxins and protect host cells.

**Figure 5:**
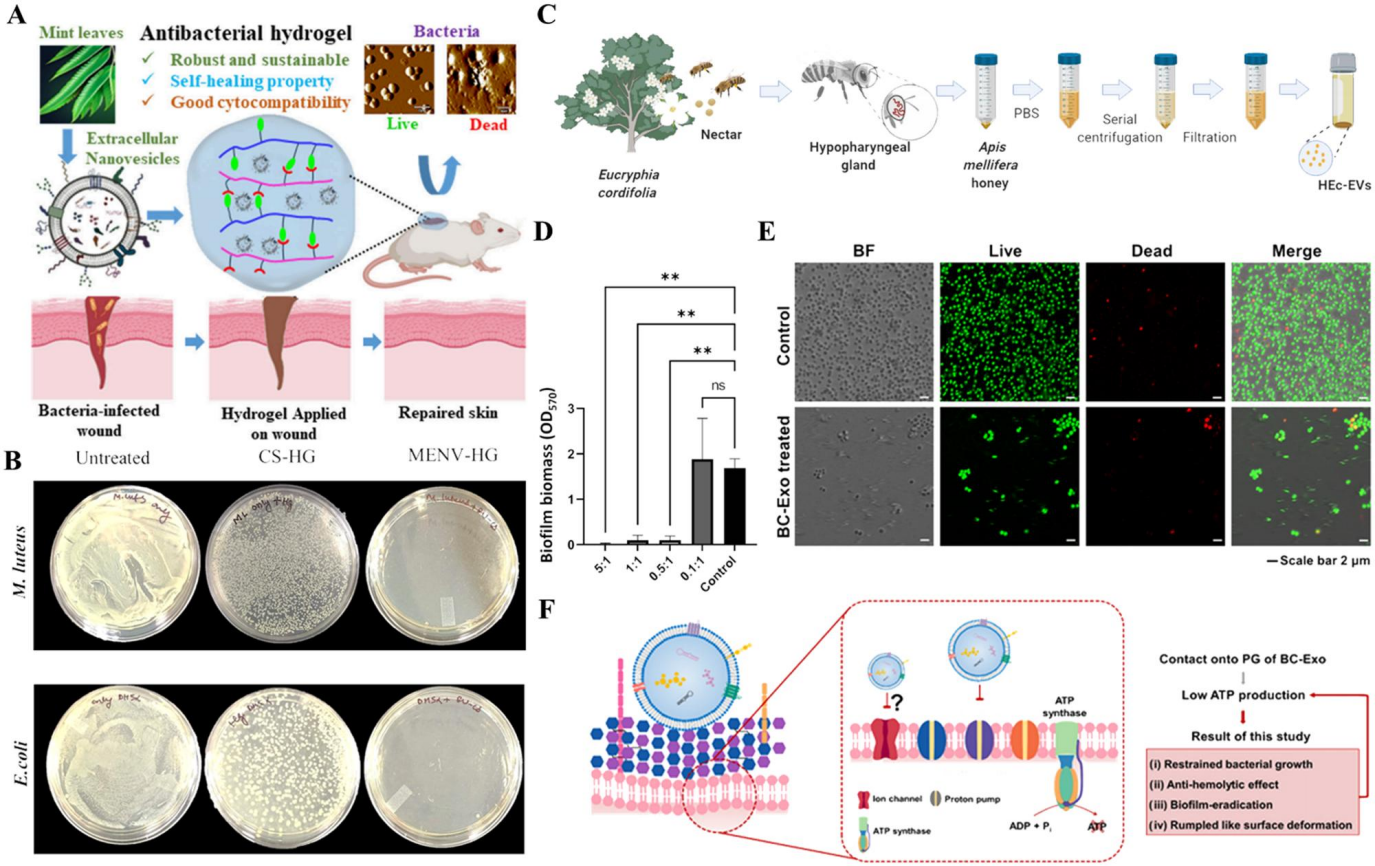
Cases of direct antibacterial use of exosomes (Note: In this figure, the symbol “*P<0.05, **P<0.015, ***P<0.0015, ****P<0.0001” indicates a highly significant statistical difference in quantitative comparisons between treated and control groups). (**A**) Fabrication of mint extracellular nanovesicle-loaded-chitosan-oxidized sodium alginate HGs (MENV-HG). (**B**) Surface antibacterial activity of MENV-HG against *M. luteus* and *Escherichia coli*; photographs of survival bacteria colony on agar plates after contact with CS-HG and MENV-HG for 3 h [92]. Copyright 2022, JoVE. (**C**) Schematic representation of HEc-EV extraction and isolation from monofloral *A. mellifera* honey utilizing ultracentrifugation (UC). (**D**) *Streptococcus mutans* UA 159. Overall, results show a differential effect of HEc-EVs against both oral streptococci, with a pronounced inhibition of *S. mutans* UA 159 growth and biofilm formation compared to *S. mutans* [84]. Copyright 2021, Leiva-Sabadini et al. (**E**) Bacteriostatic properties of BC-Exo against *Staphylococcus aureus*. Live/dead *S. aureus* treated with BC-Exo. (**F**) Bovine colostrum exosomes are a promising natural bacteriostatic agent against MSSA [83] Copyright 2023, American Chemical Society.

A primary mechanism by which exosomes exert bactericidal activity involves the destabilization of bacterial cell envelopes through direct physical and biochemical engagement. Biophysical studies suggest that this interaction is fundamentally driven by electrostatic forces. The exosomal surface is enriched with proteins bearing positively charged functional groups, such as amino and quaternary ammonium moieties, which possess a high affinity for the anionic components of the bacterial exterior, specifically lipopolysaccharides in Gram-negative bacteria and teichoic acids in Gram-positive organisms [92]. This electrostatic attraction facilitates the tight adhesion of exosomes to the bacterial surface, leading to local membrane perturbations, increased permeability and the subsequent leakage of vital intracellular contents (Figure 4A) [93–95]. Supporting this mechanism, Saroj *et al*. [92] demonstrated the intrinsic antibacterial activity of mint-derived nanovesicles against both Gram-positive *Micrococcus luteus* and Gram-negative *E. coli*, producing marked bacterial killing within 3 h *in vitro* and, when incorporated into a hydrogel, accelerating closure of bacteria-infected rat wounds with reduced bacterial growth and inflammation *in vivo* (Figure 5A and B).

Beyond nonspecific physical disruption, exosomes function as targeted delivery vectors for AMPs, thereby enhancing their lytic potential. Evolution has equipped biological fluids with vesicles carrying a potent payload of defensive molecules. For instance, extracellular vesicles isolated from honeybee products (HEc-EVs) encapsulate a diverse array of AMPs, including major royal jelly protein 1, defensin-1 and jellein-3 [84]. These peptides can promote the formation of transmembrane pores, inducing catastrophic structural collapse of the bacterial cell. *In vitro*, HEc-EVs completely inhibited *S. mutans* growth at EV:CFU ratios of 0.5:1–5:1, reduced biofilm biomass and induced AFM-detectable nanomechanical damage consistent with membrane disruption (Figure 5C and D) [96]. Similarly, bovine colostrum-derived exosomes (BC-Exo) exert their antimicrobial effects by metabolically crippling MRSA through the impairment of ATP synthesis and the concurrent degradation of cell wall integrity (Figure 5E and F) [97]. Specifically, BC-Exo showed bacteriostatic and antivirulence effects *in vitro*, with MIC50 values of 120 and 250 μg/mL in two MSSA strains, two-fold inhibition of biofilm formation, suppression of hemolysis, reduced ATP production and concurrent cell surface deformation. Consistent with this metabolism-disrupting mode of action, human umbilical cord MSC-derived apoptotic vesicles (hucMSC-ApoVs) directly inhibited and killed both MRSA and *E. coli*, accelerated infected burn-wound repair *in vivo*, and mechanistically suppressed bacterial catabolic pathways, including nucleotide and arginine metabolism in MRSA and sulfur, fatty acid, arginine/proline and ethanolamine metabolism in *E. coli* [98, 99].

Overall, current evidence indicates that exosomes and exosome-like nanovesicles can exert direct antibacterial effects through electrostatic adhesion-mediated membrane disruption, antimicrobial peptide delivery and impairment of bacterial energy metabolism and cell wall integrity. While these mechanisms are well supported by *in vitro* studies, *in vivo* validation remains relatively limited and is currently most convincing in topical infected-wound models, particularly those using plant-derived nanovesicles or hydrogel-based delivery systems. Further studies across diverse infection types, administration routes and animal models are therefore needed to confirm the robustness and mechanistic consistency of these direct antibacterial effects.

#### Gene silencing and reversal of antibiotic resistance

Perhaps the most transformative therapeutic attribute of exosomes is their ability to reverse antibiotic resistance through the targeted silencing of essential bacterial genes [100]. While bacteria lack the canonical eukaryotic RNA-induced silencing complex (RISC), recent advancements have established that exosomes can effectively deliver small interfering RNAs to manipulate bacterial gene expression, a phenomenon rooted in the evolutionary principle of cross-kingdom RNA interference (RNAi) [101].

**Figure 6:**
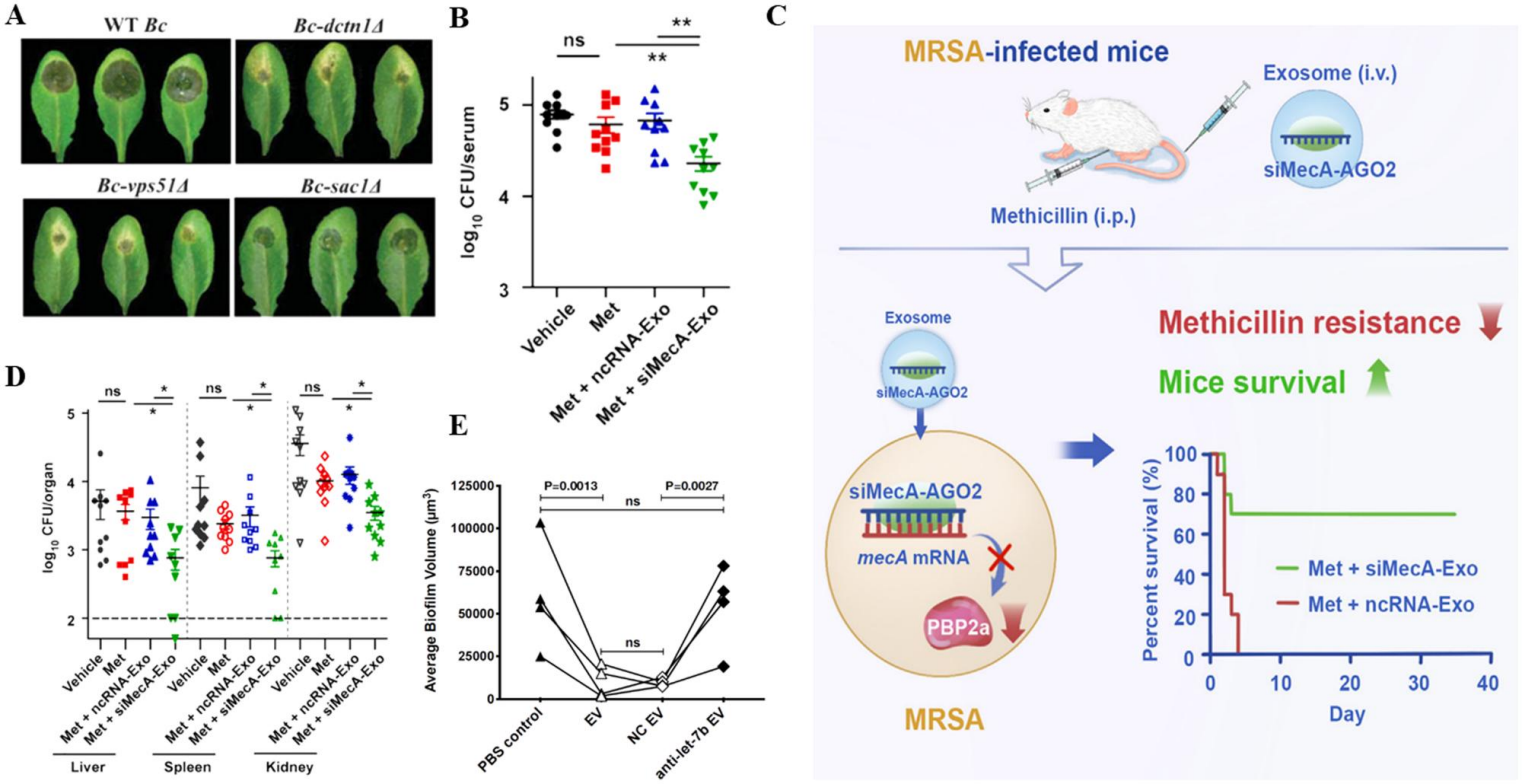
Cases of silencing bacterial RNA expression using exosomes (Note: In this figure, the symbol “*P<0.05, **P<0.015, ***P<0.0015, ****P<0.0001” indicates a highly significant statistical difference in quantitative comparisons between treated and control groups). (**A**) Mutant *B. cinerea* strains with deletions in the targets of transferred host sRNAs displayed reduced virulence. Relative lesion sizes were measured at 3 days after infection [102]. Copyright 2018, The Authors. (**B**) Effect of methicillin and siMecA-Exos on *S. aureus* survival in the sera. (**C**) siRNA-AGO2 complex inhibits bacterial gene translation: a promising therapeutic strategy for superbug infection. (**D**) Effect of methicillin and siMecA-Exos on *S. aureus* survival in the organs. (**E**) Summary of data. EVs (open triangles) reduced biofilm formation by 83% compared to *Pseudomonas aeruginosa* exposed to PBS control (filled triangles) [80]. Copyright 2021, National Academy of Sciences.

Evolutionary biology provides compelling precedents for this strategy. In the plant kingdom, small RNAs act as mobile signaling molecules that traverse plasmodesmata and vascular systems to mediate systemic defense. Crucially, Cai *et al*. [102] established host plants such as *Arabidopsis* secrete exosomes that transport sRNAs into fungal pathogens like *Botrytis cinerea*, effectively silencing virulence genes to confer disease resistance (Figure 6A). This mechanism of cross-species genetic control is conserved in mammalian systems. Wang *et al*. [103] demonstrated that exosomal Argonaute 2 protein complexes can utilize siRNAs to silence target genes through translational repression.

Leveraging this natural machinery for therapeutic purposes has yielded breakthrough results in combating drug-resistant bacteria. Researchers have successfully engineered exosomes to encapsulate specific siRNAs targeting the *mecA* gene, the molecular determinant responsible for β-lactam resistance in MRSA. The delivery of these siRNAs effectively restored the susceptibility of MRSA to methicillin both *in vitro* and in murine infection models, significantly improving survival outcomes (Figure 6B–D).

Furthermore, this gene-silencing capability extends to the disruption of bacterial biofilms, which are notoriously impervious to conventional antibiotics. Koeppen *et al*. [80] demonstrated that exosomes derived from airway epithelial cells have been shown to deliver endogenous microRNA let-7b-5p to *P. aeruginosa*. This transfer results in the downregulation of key biofilm-associated genes such as *algD* and *ndvB*, thereby dismantling the protective biofilm matrix and restoring bacterial sensitivity to β-lactam antibiotics (Figure 6E).

The direct antibacterial strategy of exosomes represents a sophisticated integration of physical, biochemical and genetic mechanisms. By combining membrane-disrupting electrostatic interactions with the delivery of pore-forming AMPs, exosomes compromise bacterial structural integrity. Simultaneously, they exploit cross-kingdom RNA interference pathways to silence critical resistance genes like *mecA*, effectively reversing drug tolerance. This multipronged attack, which further includes the disruption of protective biofilms, positions exosomes as a versatile and potent therapeutic platform capable of overcoming the multifaceted challenges posed by multidrug-resistant pathogens (Figure 4B).

### Neutralization of bacterial toxins by exosomes

The pathogenicity of bacterial infections is frequently driven not merely by bacterial burden but by the secretion of potent exotoxins that compromise host cell integrity and provoke dysregulated cytokine storms [104]. A critical limitation of conventional antibiotics is their inability to neutralize these persistent virulence factors, which often remain biologically active and continue to inflict tissue damage even after the bacteria have been eradicated [105]. To address this therapeutic gap, exosomes have been identified as employing a sophisticated molecular sequestration mechanism often described as the decoy hypothesis. In this model, exosomes function as high-affinity scavengers that intercept toxins in the extracellular milieu prior to their engagement with host cells. This neutralization capacity is mediated by the surface display of specific receptors or lipid domains that mimic the toxin’s natural cellular targets, thereby diverting the toxin away from vulnerable tissues (Figure 4C) [106, 107]. Current evidence for this mechanism is strongest at the *in vitro* level, including toxin-binding, hemolysis inhibition and cytoprotection assays, while selected studies have further validated protection in murine infection or toxin-challenge models.

**Figure 7:**
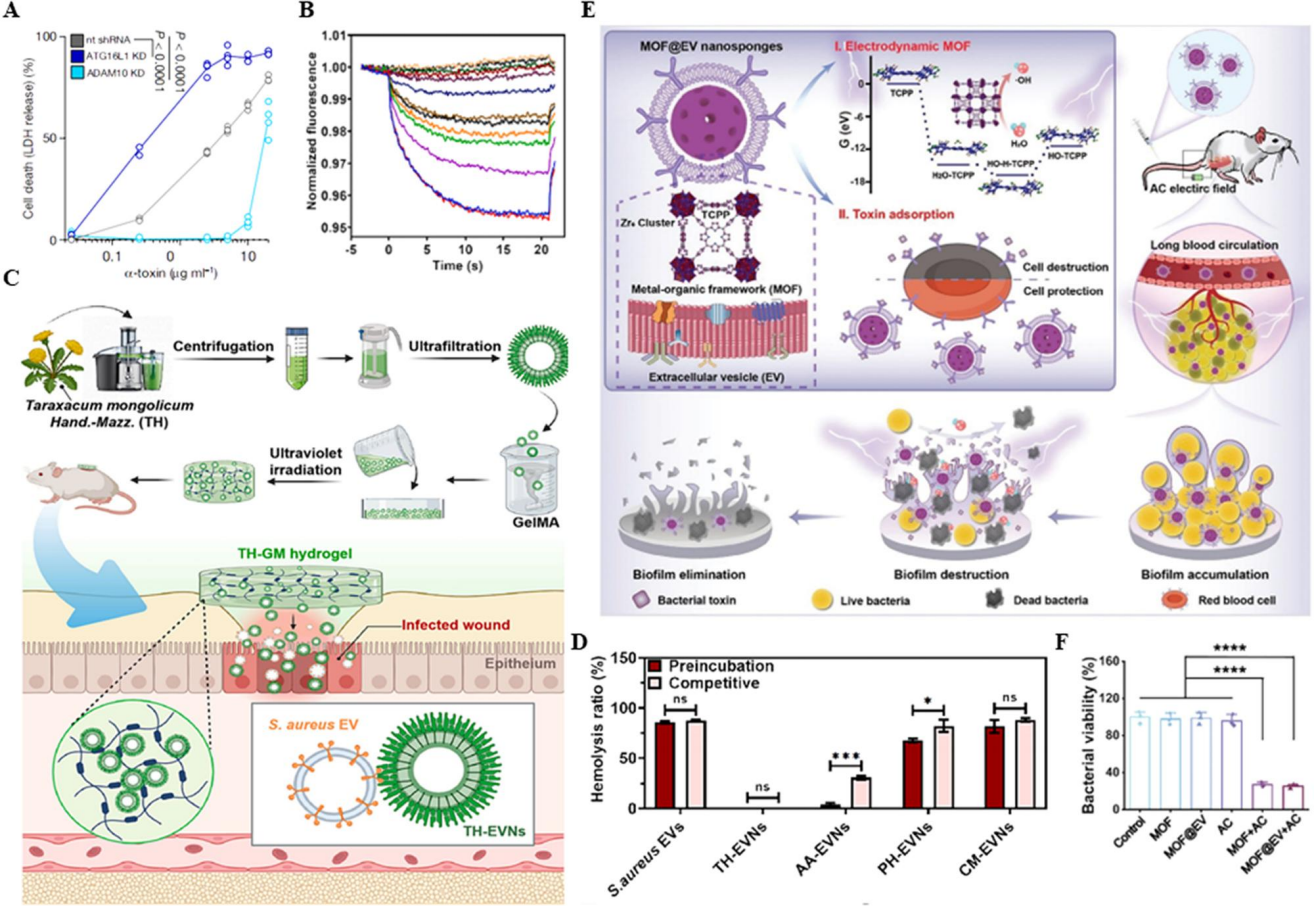
Exosomes bind to bacterial exotoxins (Note: In this figure, the symbol “*P<0.05, **P<0.015, ***P<0.0015, ****P<0.0001” indicates a highly significant statistical difference in quantitative comparisons between treated and control groups). (**A**) MST traces of FITC-labeled *S. aureus* EVs (4.9 × 10 ^− 3^ nmol/L) with different concentrations of TH-EVNs (4.7 × 10 ^− 5^ -1.55 nmol/L). (**B**) Quantification of cell death (assayed by release of lactate dehydrogenase) of nt shRNA, ATG16L1 KD and ADAM10 KD cells following treatment with purified α-toxin; *n* = 4 [108]. Copyright 2020, Springer Nature Limited. (**C**) Schematic illustrations of photocrosslinked hydrogel loaded with dandelion-derived extracellular vesicle-like nanoparticles for *S. aureus* EVs-invasive wound. (**D**) Hemolysis quantification of four EVNs after incubation with *S. aureus* EVs were evaluated in both preincubation and competitive regimens [109]. Copyright 2024, Elsevier B.V. (**E**) Schematic illustration of the preparation of MOF@EV and its antibacterial mechanism. (**F**) Bacterial viability (*n* = 3) [112]. Copyright 2024, The Author(s).

This molecular interception strategy is powerfully exemplified by the interaction between mesenchymal stem cell-derived exosomes and the pore-forming toxins of *S. aureus*. Specifically, these exosomes effectively neutralize the α-toxin, a key virulence factor responsible for host cell lysis. Building upon foundational research that established the autophagy protein ATG16L1 as a critical component of intracellular defense, Keller *et al*. elucidated a novel extracellular protection mechanism [108]. Their investigation revealed that exosomes are naturally enriched with ADAM10, the specific cellular receptor for the α-toxin, alongside autophagy-associated proteins. By presenting ADAM10 on their surface, these exosomes act as competitive inhibitors that bind the α-toxin with high affinity (Figure 7A) [108]. This interaction competitively inhibits toxin engagement with cellular ADAM10 receptors, thereby preventing pore assembly on host membranes and subsequent cytolysis. Significantly, this work demonstrates a sophisticated evolutionary adaptation whereby exosomes extracellularly deploy defense machinery to systemically neutralize virulence factors, complementing traditional intracellular autophagy pathways. Importantly, this work extended beyond cell culture: transfer of exosomes induced by bacterial components improved survival in MRSA-infected mice, supporting the *in vivo* relevance of the decoy mechanism [108].

Expanding the repertoire of vesicle-based therapeutics, recent investigations have highlighted the potential of plant-derived nanoplatforms to execute similar toxin-neutralizing functions. Unlike mammalian systems, plant-derived extracellular vesicles offer distinct advantages in terms of scalability, stability and the absence of zoonotic risks. Tan *et al*. [109] demonstrated that vesicle-like nanoparticles isolated from *Taraxacum officinale* possess potent neutralizing activity against *S. aureus* α-hemolysin (Figure 7B and C). These bioactive nanoparticles utilize a rich cargo of specific lipids and proteins to sequester the toxin with nanomolar affinity (Figure 7D). *In vitro*, dandelion-derived extracellular vesicle-like nanoparticles (TH-EVNs) reduced *S. aureus* EV-induced hemolysis from approximately 80% to 4%, and their activity was maintained under saline, serum, and plasma conditions. In mice, they reduced toxin-induced skin lesions, apoptosis and inflammatory cytokines, including TNF-α, IL-1β and IL-6. This robust binding irreversibly inhibits the hemolytic activity of the toxin *in vitro* and significantly attenuates inflammation and tissue necrosis in murine infection models [110].

Critically, this approach represents a paradigm shift toward antivirulence therapy. By selectively targeting the bacterial weapon rather than the bacterium itself, plant-derived vesicles avoid imposing the selective pressure that drives antibiotic resistance. Furthermore, this strategy preserves the integrity of the commensal microbiota, a frequent casualty of broad-spectrum antibiotic treatment. The ability of these plant-derived vesicles to function as broad-spectrum toxin sponges without disrupting the ecological balance of the host highlights their potential as a sustainable alternative for managing toxin-mediated diseases [111]. However, because vesicle-only toxin neutralization does not necessarily reduce bacterial burden or biofilm biomass, it may be most appropriate as an adjunctive strategy in toxin-driven inflammation, invasive wounds or post-antibiotic tissue protection [111].

Inspired by these natural defense mechanisms, advanced bioengineering strategies have emerged to enhance the sequestration capacity of vesicles through the integration of synthetic nanomaterials. Wang *et al*. [112] developed a biomimetic metal-organic nanosponge by fusing ginger-derived extracellular vesicles with electrodynamic metal-organic frameworks (Figure 7E). This hybrid platform exploits the synergistic properties of its components. The ginger vesicle membrane provides biological stability and prolongs the circulatory half-life of the construct, facilitating its accumulation at the site of infection. Simultaneously, the metal-organic framework core offers a high surface area for the adsorption of diverse bacterial virulence factors. This engineered system functions as a dual-action therapeutic that not only blocks the specific binding of toxins to host cells but also broadly adsorbs circulating inflammatory mediators (Figure 7F) [112]. Compared with vesicle-only or hydrogel-retention strategies, MOF@EV combined toxin sequestration with electrodynamic ROS generation, thereby addressing both virulence factors and bacterial/biofilm burden. *In vitro*, MOF@EV plus an AC electric field achieved approximately 99% killing of planktonic *S. aureus* and over 99.9% killing in biofilm assays while reducing hemolytic activity and restoring toxin-impaired L929 cell viability. In a murine subcutaneous abscess model, MOF@EV plus AC markedly decreased bioluminescent bacterial signals, viable bacterial counts, neutrophil infiltration, MPO staining and inflammatory cytokine [112]. By comprehensively reducing the toxic burden in the microenvironment, such hybrid systems demonstrate superior therapeutic efficacy compared to single-mechanism approaches, effectively mitigating cellular damage and accelerating recovery.

In summary, the utilization of exosomes as toxin-neutralizing agents constitutes a transformative approach in anti-infective therapeutics. Whether derived from mammalian stem cells, medicinal plants or engineered hybrid systems, these vesicles function as effective molecular decoys that overcome the fundamental limitations of antibiotics. By physically sequestering pore-forming toxins and preventing their interaction with host tissues, exosome-based platforms concurrently address pathogen virulence and host protection. This integrated capability, combined with their inherent biocompatibility and potential for encapsulation in advanced delivery matrices like hydrogels, heralds a promising therapeutic frontier. Future research focusing on the scalable manufacturing of these decoys and their validation in complex polymicrobial models will be essential for translating this antivirulence strategy into clinical practice (Figure 7).

### Immunomodulatory functions of exosomes

The progression and ultimate resolution of bacterial infections are contingent upon the plasticity of macrophages, which must dynamically transition between a pro-inflammatory M1 phenotype and an anti-inflammatory M2 state. Exosomes have emerged as pivotal orchestrators of this phenotypic flexibility, delivering molecular instructions that fine-tune the immune response to align with the specific stage of infection [113]. Crucially, this regulatory network is dictated by strict cell-type-specific dynamics within the highly compartmentalized infection microenvironment, where structural cells, innate immune responders and adaptive lymphocytes utilize customized exosomal messages. The cellular origin of the exosome determines its unique cargo composition, and its downstream therapeutic or pathological effects are tightly governed by the specialized receptor profiles and spatial distribution of the recipient cells [114].

During the acute phase, the immunological priority is pathogen eradication. Exosomes derived from various immune sentinels coordinate to drive macrophages toward the bactericidal M1 phenotype, illustrating a sophisticated division of labor among distinct donor cell types [115]. Initiating this cascade, dendritic cells secrete exosomes enriched with miR-155. Upon internalization by naïve macrophages, this specific microRNA targets and silences the Suppressor of Cytokine Signaling 1, a negative regulator of the JAK-STAT pathway. The removal of this checkpoint disinhibits STAT1 signaling, thereby amplifying the production of essential pro-inflammatory cytokines, including TNF-α, IL-6 and IL-12 [116]. This mechanism is synergistically reinforced by NK cell-derived exosomes, which deliver both miR-155 and IFN-γ. While dendritic cell exosomes primarily modulate the JAK-STAT checkpoint via microRNA transfer, NK-derived vesicles directly supply the canonical cytokine stimulus (IFN-γ). This highlights a clear dichotomy between myeloid- and lymphoid-derived exosomes: the former are often predisposed to antigen presentation circuitry and checkpoint regulation, whereas the latter excel in remote phenotype enforcement through specialized effector cytokines [117]. These cargoes activate the STAT1-IRF5 axis to upregulate inducible nitric oxide synthase, significantly enhancing the oxidative killing capacity of macrophages [118].

**Figure 8:**
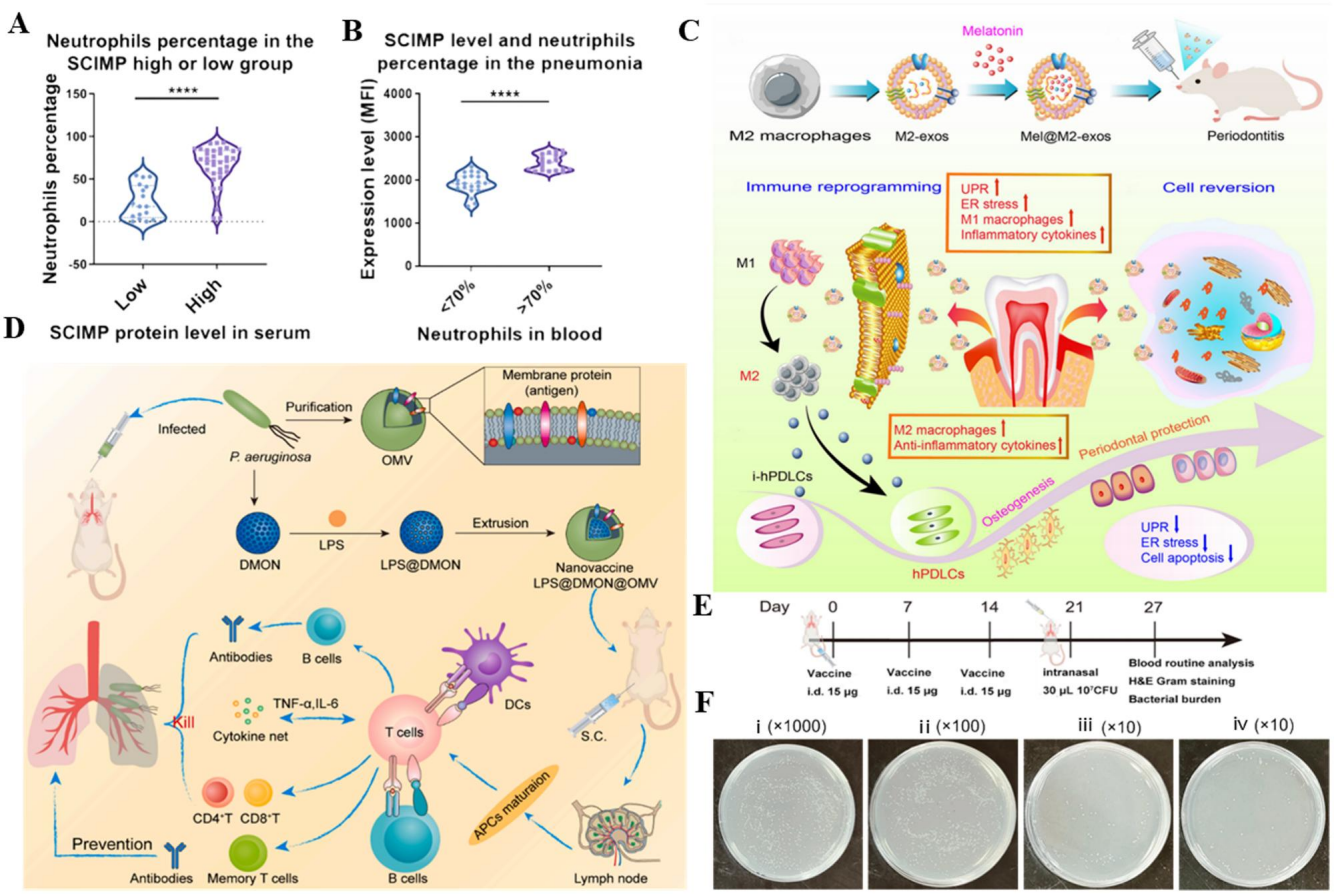
Exosomes regulate immune function against bacterial infection (Note: In this figure, the symbol “*P<0.05, **P<0.015, ***P<0.0015, ****P<0.0001” indicates a highly significant statistical difference in quantitative comparisons between treated and control groups). (**A**) The serum samples from the pulmonary-infected patients were subgrouped according to the exosomal SCIMP expression level in the serum (>2000, *n* = 40 or <2000, *n* = 17) and the neutrophils percentage in the white blood cells in these two subgroups was measured and analyzed. (**B**) The serum samples from the pulmonary-infected patients were subgrouped based on the neutrophil percentage in the white blood cells (>70%, *n* = 30 or <70%, *n* = 27), and the exosomal SCIMP expression level of the serum samples in these two subgroups was measured and analyzed [120]. Copyright 2024, The Author(s). (**C**) Schematic representation of engineered M2 macrophage-derived exosomes (M2-Exos) for treating inflammatory bone loss in periodontitis through mediating ER stress and immune reprogramming [124]. Copyright 2023, The Author(s). (**D**) A schematic diagram of the antibacterial immune regulation of nanovaccine encapsulated by bacterial OMVs. (**E**) Timeline of the experimental design to evaluate the antibacterial infection experiment in immunized mice. (**F**) Bacterial burden in the lungs of mice treated with different methods [126]. Copyright 2022, Elsevier B.V.

Beyond intracellular signaling, exosomes function as intercellular “distress beacons” that propagate immunity to bystander cells, mediated by distinct myeloid-lineage interactions. In the context of *M. tuberculosis* infection, macrophages act as amplifying vectors; infected cells release exosomes loaded with PAMPs and host-derived danger signals that prime neighboring uninfected macrophages for heightened surveillance. A pivotal discovery in this domain is the identification of the SLP adapter and CSK-interacting membrane protein (SCIMP) as a unique exosomal cargo [119]. Notably, Pei *et al*. [120] revealed that exosomal SCIMP exists in a structurally distinct, N-terminally truncated form. This modification endows the protein with a novel biological function as a potent neutrophil chemoattractant, operating independently of classical formyl peptide receptor signaling. By utilizing this alternative recruitment pathway, macrophage exosomes effectively bridge the gap between mononuclear phagocytes and granulocytes, mobilizing neutrophils to infectious foci to execute rapid bacterial clearance (Figure 8A and B). This phenomenon underscores the critical importance of recipient cell selectivity; the functional outcome of exosomal delivery relies entirely on the unique layout of downstream signaling partners on the recipient cell type, allowing macrophage-derived vesicles to target neutrophils specifically rather than other neighboring lymphocytes.

Importantly, this cell-type-specific communication cascade is not restricted to professional hematopoietic cells. Nonimmune structural cells, such as epithelial and endothelial cells at the mucosal barrier, are the first to encounter pathogens [121]. Their distinct exosomal profiles—characteristically enriched with localized defense molecules like mucins, antimicrobial peptides and tissue-specific miRNAs—serve to alert resident alveolar or tissue macrophages long before professional immune sentinels are recruited to the niche [122]. While the initial inflammatory burst is essential for defense, its timely resolution is critical to prevent collateral tissue damage and chronic immunopathology. As infection subsides, the immunological landscape must shift toward homeostasis. Exosomes facilitate this transition by driving macrophage repolarization from the M1 “destroyer” phenotype to the M2 “healer” phenotype, characterized by the secretion of anti-inflammatory mediators such as IL-10 and TGF-β [123]. This restorative function is particularly vital in chronic inflammatory conditions, such as periodontitis. Therapeutic strategies have successfully harnessed this natural switching mechanism; for instance, exosomes engineered from melatonin-treated macrophages (Mel@M2-exos) have demonstrated a remarkable capacity to resolve periodontal inflammation. These vesicles function through a dual mechanism: they not only induce the phenotypic switch to M2 but also actively mitigate cellular stress responses. Specifically, they alleviate ER stress and suppress the unfolded protein response (UPR), thereby reducing apoptotic cell death and creating a microenvironment conducive to osteogenesis and soft tissue regeneration (Figure 8C) [124]. Importantly, this strategy has moved beyond cell culture: sustained release of Mel@M2-exos from injectable GelMA hydrogels enhanced local retention, reduced inflammatory destruction and accelerated periodontal bone regeneration in ligation-induced rat periodontitis, supporting hydrogel-assisted exosome delivery as a promising combination strategy [124].

Complementing the immunomodulatory roles of host-derived exosomes, BEVs offer a unique avenue for proactive immune education, acting as cell-type-specific triggers for antigen-presenting cells. Bacterial OMVs are naturally immunogenic nanostructures that present native bacterial antigens in their physiological conformation. When recognized by pattern recognition receptors on host immune cells, these vesicles trigger potent innate defense mechanisms (Figure 8D) [125]. Recent bioengineering advances have transformed these natural vesicles into sophisticated nanovaccine platforms. By engaging TLR4 and CD14 receptors on dendritic cells, engineered OMV-based vaccines stimulate robust dendritic cell maturation and antigen presentation. In pneumonic infection models, such platforms have been shown to elicit durable Th1 and Th17 adaptive immune responses, leading to the generation of high-titer pathogen-specific antibodies and the establishment of long-term immunological memory (Figure 8E and F) [126]. Ultimately, moving past treating “exosomes” as a single homogenous functional class and instead deciphering the complete interactome of specific donor-to-recipient cell pairs will be vital. Mapping this cell-type-specific code within the infection microenvironment represents a crucial frontier for designing targeted, bioengineered exosomal therapies capable of intervening in precise cellular communications without causing off-target immunopathology.

**Figure 9:**
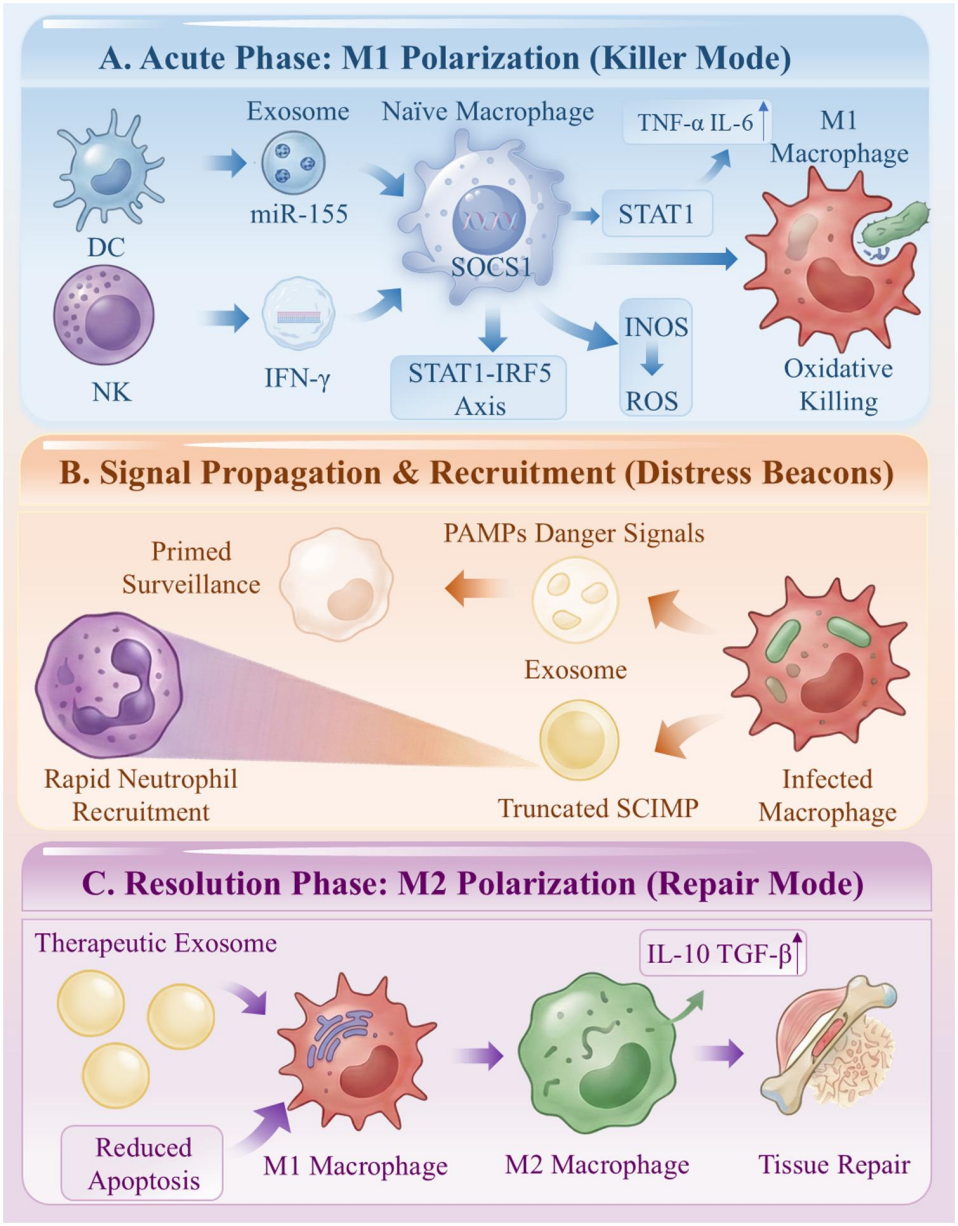
Exosomes regulate immune functions against bacterial infection. This schematic illustrates the spatiotemporal immunomodulatory roles of exosomes in combating bacterial infections. (**A**) Exosome-driven M1 macrophage polarization for acute oxidative bacterial killing; (**B**) the propagation of exosomal distress beacons for rapid neutrophil recruitment; (**C**) therapeutic exosome-mediated reprogramming of macrophages toward an M2 phenotype to resolve inflammation and promote tissue repair.

Collectively, the immunomodulatory landscape of exosomes represents a sophisticated continuum of biological control. Host-derived exosomes act as dynamic switches that initiate the necessary inflammatory attack through M1 polarization and SCIMP-mediated neutrophil recruitment, subsequently orchestrating the resolution phase via M2 repolarization and stress mitigation. Concurrently, bacterial-derived vesicles provide the antigenic templates required to train the adaptive immune system. The integration of these complementary mechanisms—harnessing the intrinsic regulatory power of host exosomes and the immunogenicity of bacterial vesicles—creates a versatile foundation for the development of next-generation immunotherapeutic (Figure 9).

### Antibacterial drug carriers by exosomes

**Figure 10:**
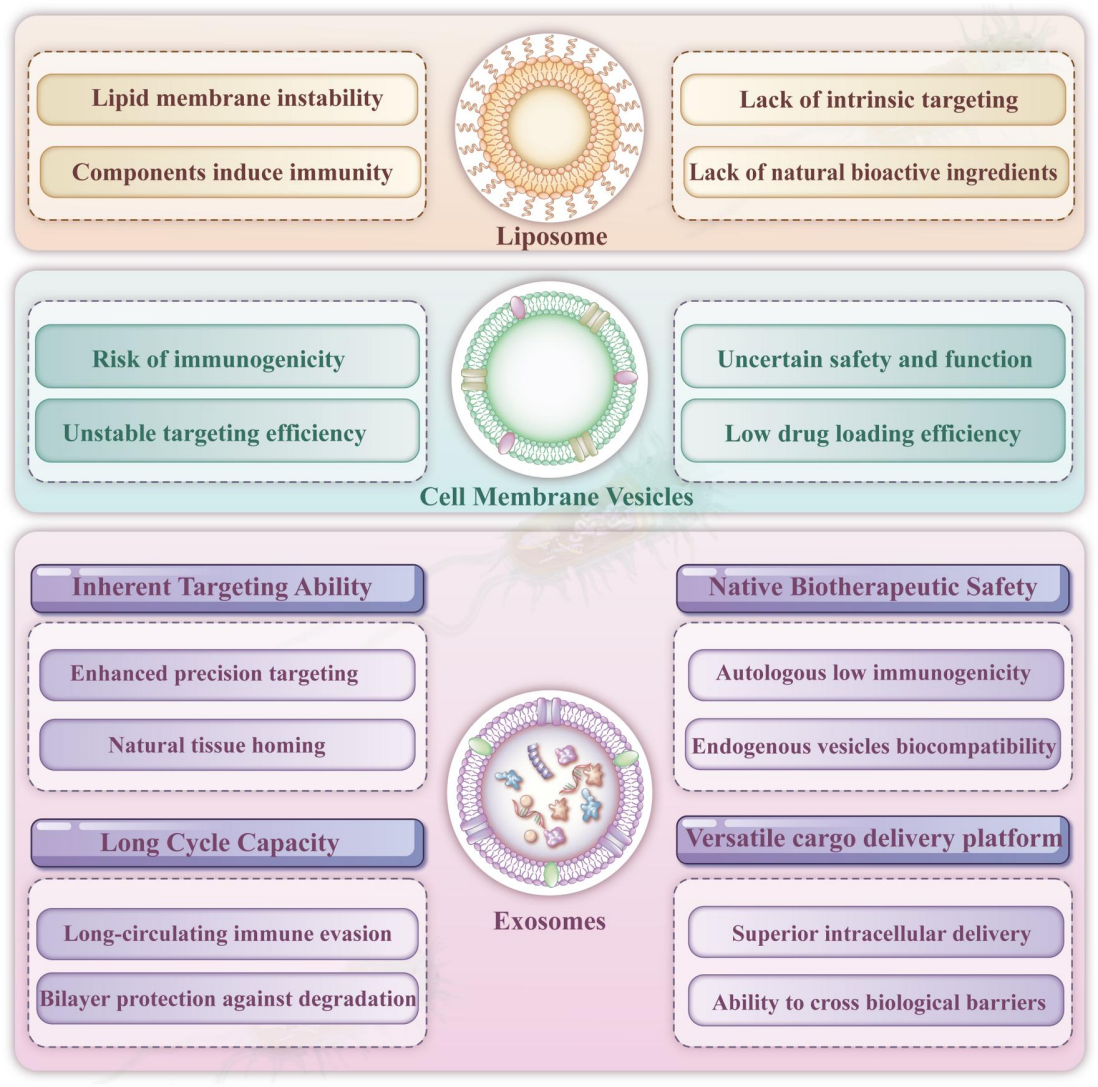
Comparison of the advantages and disadvantages of liposomes, cell membrane vesicles and exosomes as drug carriers in the treatment of bacterial infections.

The paradigm of antimicrobial drug delivery is currently witnessing a transition from synthetic liposomes to naturally derived exosomes. This shift is driven by the superior intrinsic properties of exosomes, including their exceptional biocompatibility, minimal immunogenicity and inherent capacity to traverse physiological barriers, such as the gastrointestinal mucosa. Unlike synthetic counterparts, exosomes possess a sophisticated lipid-protein membrane composition that facilitates cellular uptake and organ-specific accumulation, positioning them as transformative tools for enhancing the therapeutic index of conventional antibiotics [127]. Among the various sources, bovine milk exosomes have emerged as leading candidates for clinical translation due to their nutritional ubiquity, lack of toxicity and the potential for scalable, cost-effective production. Complementary research has also highlighted camel milk exosomes as a distinct reservoir, offering unique lipid profiles that may further enhance antimicrobial stability (Figure 10) [128].

**Figure 11:**
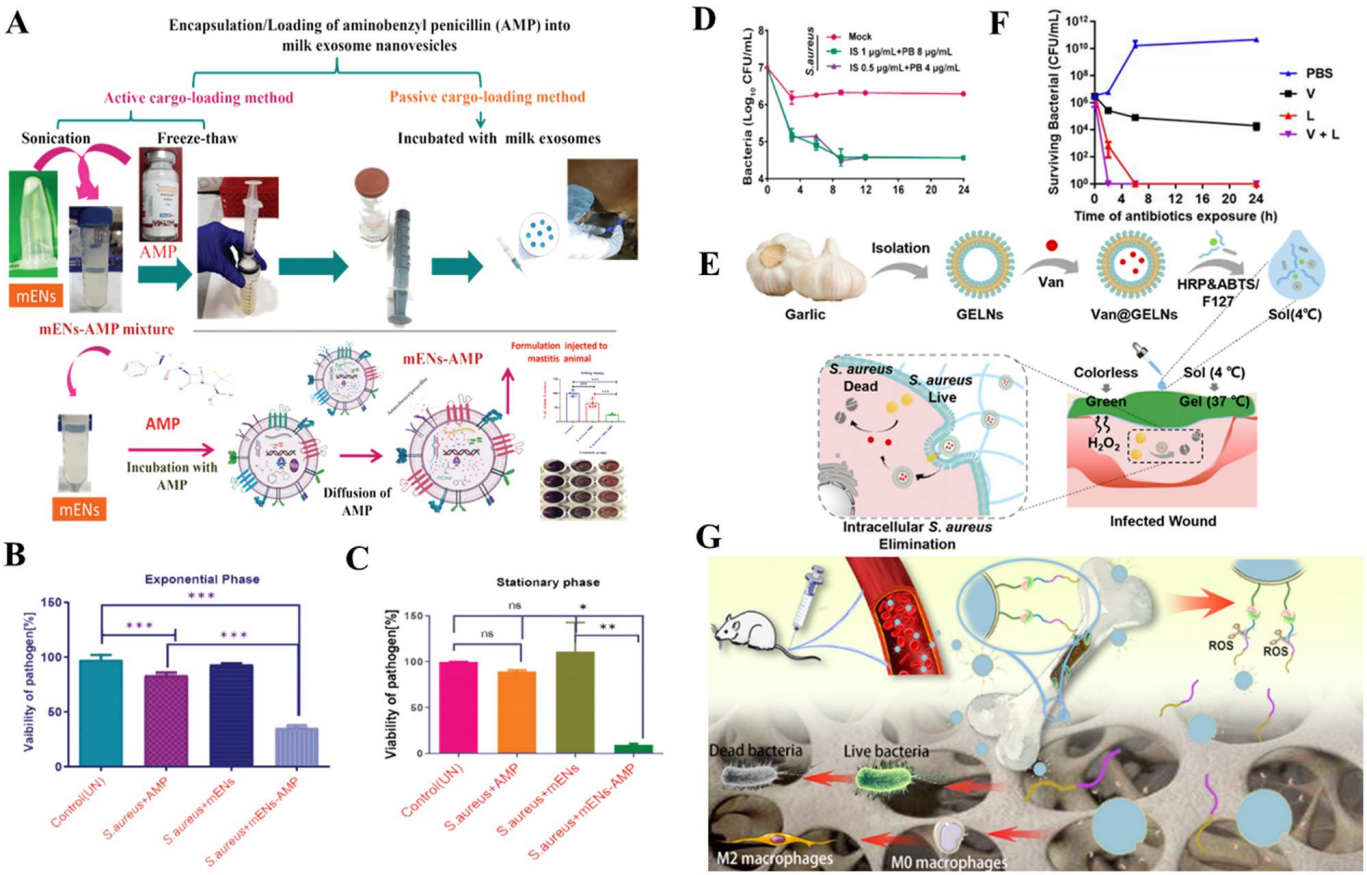
Exosomes as a delivery system for combined antibacterial bacteria (Note: In this figure, the symbol “*P<0.05, **P<0.015, ***P<0.0015, ****P<0.0001” indicates a highly significant statistical difference in quantitative comparisons between treated and control groups). (**A**) Schematic illustrations for the development of aminobenzylpenicillin-encapsulated exosome nanovesicle preparation. Graphical images were created with biorender.com. (**B**, **C**) *In vitro* antibacterial and killing kinetic assays were performed. *S. aureus* viability after treatment included a blank (BHI/BPA), control (untreated) *S. aureus*, *S. aureus* + mENs, *S. aureus* + AMP and *S. aureus* + mENs-AMP [129]. Copyright 2024, Wiley-VCH GmbH. (**D**) Time-fungicidal curves of different treatment groups against *S. aureus* [128]. Copyright 2022, Elsevier B.V. (**E**) GELNs with antimicrobial activity were extracted from garlic, and Van@GELNs were obtained after Van loading [70]. Copyright 2025, Elsevier B.V. (**F**) Time of kill for free antibiotics on nonreplicating MRSA [131]. (**G**) Schematic of engineered RAB-EXO treatment for osteomyelitis [132]. Copyright 2024, Royal Society of Chemistry.

Initial therapeutic strategies focused on the encapsulation of single agents to optimize their pharmacokinetic profiles. Kumar *et al*. [128] validated this approach by loading bovine milk exosomes with AMPs to treat MRSA-induced mastitis. The exosome-encapsulated peptides demonstrated significantly higher efficacy in reducing bacterial loads compared to free peptides, a result attributed to the vesicle’s ability to enhance epithelial permeability and protect the payload from enzymatic degradation (Figure 11A–C). Specifically, this study provided both *in vitro* and *in vivo* evidence: mENs-AMP showed approximately 11-fold higher antibacterial activity and a fourfold lower MIC than free AMP *in vitro*, while in mastitis-affected cows it significantly reduced *S. aureus* load and somatic cell counts, indicating concurrent antibacterial and inflammation-relieving effects [128]. Advancing beyond monotherapy, recent innovations have addressed the challenge of polymicrobial resistance through combinatorial loading. Researchers have successfully co-encapsulated isobavachalcone and polymyxin B within bovine milk exosomes to create a dual-action system. This formulation achieved synergistic bacterial killing through a complementary mechanism where isobavachalcone induced lipid erosion of the bacterial membrane while polymyxin B triggered structural collapse. This concerted attack not only eradicated pathogens but also accelerated wound healing in infected tissue models (Figure 11D) [129]. However, the translation of milk exosomes is currently hindered by technical challenges, specifically the interference of casein micelles during isolation, which complicates purification. Overcoming this separation hurdle remains a critical prerequisite for harnessing the full clinical potential of this abundant resource.

Transitioning from mammalian to botanical systems, plant-derived ELNs represent a unique class of carriers that function not merely as inert vehicles but as bioactive scaffolds. These vesicles offer superior stability in harsh environmental conditions and possess a high loading capacity for hydrophilic and hydrophobic molecules. Crucially, many ELNs, such as those derived from garlic (*Allium sativum*), are naturally enriched with defense-associated metabolites like allicin and quercetin, which exhibit intrinsic antibacterial activity [130]. This inherent bioactivity allows for the design of “carrier-drug” synergistic therapies. By loading garlic ELNs with vancomycin, Zhou *et al*. [70] engineered a dual-action nanomedicine that significantly enhanced antibacterial efficacy. The mechanism involves the additive effect of the vesicle’s natural antimicrobial components and the synthetic antibiotic, resulting in prolonged systemic circulation and the targeted elimination of *S. aureus* in murine infection models (Figure 11E). This strategy effectively lowers the required dose of antibiotics, thereby reducing systemic toxicity.

While natural exosomes offer improved bioavailability, the eradication of intracellular bacteria that evade antibiotics by hiding within host cells necessitates advanced engineering strategies. These “smart” exosome systems are designed to actively target specific cell types or respond to pathological microenvironments.

To dismantle intracellular reservoirs of MRSA within macrophages, Yang *et al*. [131] employed a metabolic labeling strategy combined with bioorthogonal click chemistry. By incorporating azide-modified lipids into the exosome membrane and subsequently conjugating them with mannose ligands, they created a “Trojan Horse” delivery system. These mannose-functionalized exosomes were actively recruited by mannose receptor-positive macrophages, hijacking the natural immune trafficking pathways to deliver high concentrations of vancomycin and lysostaphin directly into the infected cytosol. This precise intracellular accumulation reduced the effective antibiotic dose by 80% while minimizing off-target side effects (Figure 11F). The supporting evidence mainly includes macrophage intracellular infection models and MRSA-infected mice, where the combined delivery of vancomycin and lysostaphin showed superior clearance of intracellular, especially quiescent, MRSA compared with single-drug systems [131].

Expanding this precision approach to complex anatomical niches such as bone infections, Chen *et al*. developed a stimuli-responsive system for osteomyelitis [132]. They engineered exosomes surface-modified with reactive oxygen species (ROS)-cleavable multifunctional peptides. These designed vesicles (RAB-EXO) achieved a sequential therapeutic effect. First, they facilitated efficient uptake by macrophages and induced polarization toward the anti-inflammatory M2 phenotype to resolve tissue damage. Subsequently, upon encountering the high ROS environment typical of the infection site, the surface peptides were cleaved, triggering the on-demand release of antimicrobial payloads to eradicate pathogens like MRSA and *E. coli* (Figure 11G) [132]. This conclusion is supported by both *in vitro* antibacterial and antibiofilm assays and an *in vivo* rat osteomyelitis model. RAB-EXO inhibited MRSA/*E. coli*, disrupted biofilm formation, reduced inflammatory cell infiltration and pro-inflammatory cytokine expression, promoted M2 macrophage polarization and achieved bone repair within 28 days. Therefore, intracellular MRSA infection and osteomyelitis currently represent relatively well-supported application scenarios for engineered exosome carriers [132].

Despite these advances in potency and targeting precision, the translational application of engineered exosomes still requires a careful assessment of nontarget effects and safety liabilities. Surface engineering strategies, including click chemistry, ligand conjugation, peptide decoration and polymer modification, can improve lesion homing and cellular uptake, but they may also alter the native biodistribution, clearance route and protein corona of exosomes [133]. Consequently, engineered vesicles may accumulate in reticuloendothelial or filtration organs such as the liver, spleen, kidney and lung, leading to unintended exposure of healthy tissues to antibiotics, antimicrobial peptides or bioactive membrane ligands. Such off-target deposition could narrow the therapeutic window by inducing local cytotoxicity, mitochondrial stress, membrane damage or organ-specific toxicity, particularly when repeated administration or high surface ligand density is required [134].

In addition, the immunological consequences of exosome engineering remain insufficiently defined. Although native exosomes are generally considered biocompatible, exogenous targeting moieties, synthetic linkers, PEGylated components or non-native peptides may be recognized by host immune surveillance systems and modify macrophage or dendritic-cell responses [135]. In susceptible inflammatory microenvironments, these modifications may promote NF-κB activation, inflammasome priming or excessive secretion of TNF-α, IL-1β and IL-6, thereby counteracting the intended anti-inflammatory function of the carrier. Therefore, future studies should evaluate engineered exosomes not only by antibacterial efficacy but also by biodistribution, organ retention, dose-dependent cytotoxicity, cytokine profiles, complement activation and long-term inflammatory outcomes in relevant infection models [136].

The development of exosome-based carriers represents a significant evolution in antimicrobial pharmacotherapy. The field is advancing from the use of simple, naturally abundant milk exosomes to bioactive plant-derived vesicles and finally to sophisticated, engineered systems capable of spatiotemporal control. These platforms integrate the principles of material science with biological specificity, offering unique capabilities to penetrate anatomical barriers, target intracellular niches and respond to microenvironmental cues. By simultaneously neutralizing pathogens and modulating host immune responses, these next-generation nanomedicines provide a multifaceted solution to the limitations of conventional antibiotics. However, unlocking their full clinical potential will ultimately depend on a more balanced approach—one that strictly evaluates and mitigates off-target accumulation, cellular toxicity and host pro-inflammatory risks alongside their direct antimicrobial efficacy.

## Exosome-based combination therapy against bacterial infections

Within the global public health domain, the threat of bacterial infections has intensified due to the rapid proliferation of antibiotic-resistant pathogens. Conventional monotherapy often proves inadequate against multidrug-resistant bacteria such as MRSA and resistant Gram-negative species [137]. In this context, exosomes—nanoscale extracellular vesicles actively secreted by cells—have emerged as promising therapeutic agents, leveraging their innate biological properties. As natural mediators of intercellular communication, exosomes exhibit high biocompatibility, low immunogenicity and a remarkable capacity to traverse biological barriers. However, realizing their full clinical potential requires overcoming key challenges, including achieving controlled, sustained release, ensuring effective accumulation at target sites and integrating synergistic physical antibacterial mechanisms [138]. This issue is particularly relevant because exosomes generally exhibit a short *in vivo* half-life and can be rapidly cleared by phagocytic cells, which limits their ability to maintain effective concentrations in infected tissues [139]. Therefore, for infections with accessible or well-defined lesions, local administration may be more advantageous than systemic delivery, as it can enhance lesion-site retention, reduce systemic clearance and minimize off-target exposure [140]. In contrast, systemic administration may be more appropriate for disseminated infections or sepsis, but it requires additional strategies to improve circulation stability, tissue targeting and dose controllability.

**Figure 12:**
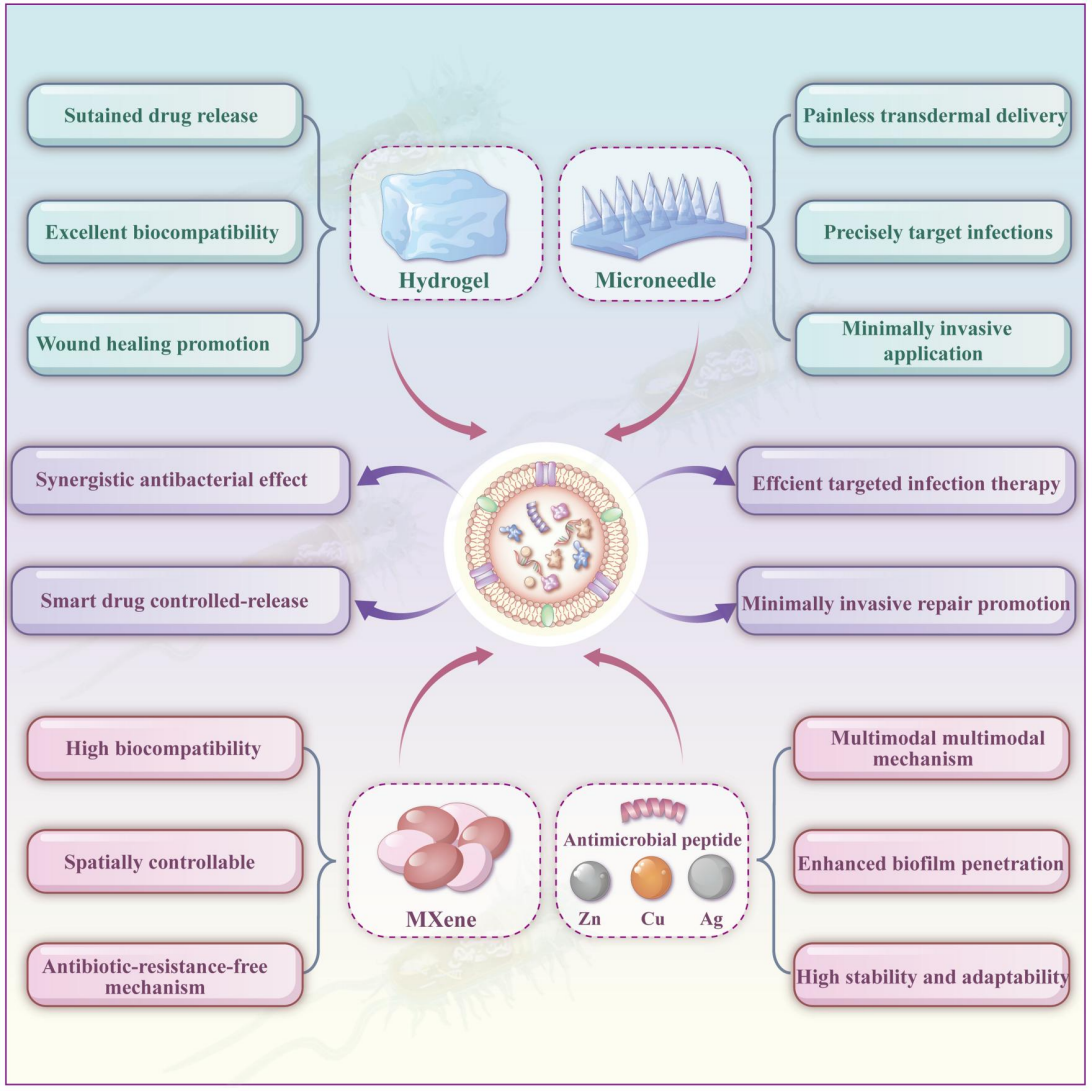
Exosomes combined with hydrogels, microneedles and functional materials jointly combat bacterial infections.

To address these limitations, the convergence of exosome biology with advanced materials science and engineering has given rise to innovative combinatorial platforms, marking a promising frontier for next-generation anti-infective strategies (Figure 12). In this regard, biomaterial-based delivery systems, including hydrogels, injectable scaffolds, microneedle patches and nanofiber membranes, can function as local depots that prolong exosome retention, enable sustained release and protect vesicles from premature degradation. For systemic use, surface engineering strategies such as PEGylation, CD47-mediated antiphagocytic modification or ligand-mediated targeting of inflamed endothelium and infected tissues may help reduce rapid clearance and improve lesion accumulation. Importantly, in these combination systems, the therapeutic outcome is not determined solely by the biological activity of exosomes or the antibacterial effect of the auxiliary component but also by key biomaterial parameters such as pore size, degradation kinetics and mechanical strength. These structural and physicochemical features govern exosome loading, diffusion, retention, protection from premature inactivation and the ability of the scaffold to maintain intimate contact with infected tissues, thereby directly shaping anti-infective efficacy and subsequent tissue regeneration.

### Exosome-based antibiotic delivery therapy

While exosomes possess immense therapeutic potential, their clinical translation is hindered by rapid clearance, poor retention at target sites and instability in physiological environments. To overcome these barriers, hydrogels have emerged as ideal scaffolding materials. Their porous architecture, high water content and tunable physicochemical properties allow them to serve as protective reservoirs that prolong the residence time of exosomes and facilitate their sustained, spatiotemporal release [141]. Moreover, the hydrogel matrix itself can be engineered to possess intrinsic biological activities, such as promoting cell adhesion or inhibiting bacterial colonization, thereby creating a synergistic platform for infection control [142]. Compared with free exosomes or free antimicrobial agents, hydrogel-assisted delivery is especially advantageous for localized infections because it improves cargo retention, reduces premature diffusion and enables simultaneous antibacterial, anti-inflammatory and tissue-reparative effects.

**Figure 13:**
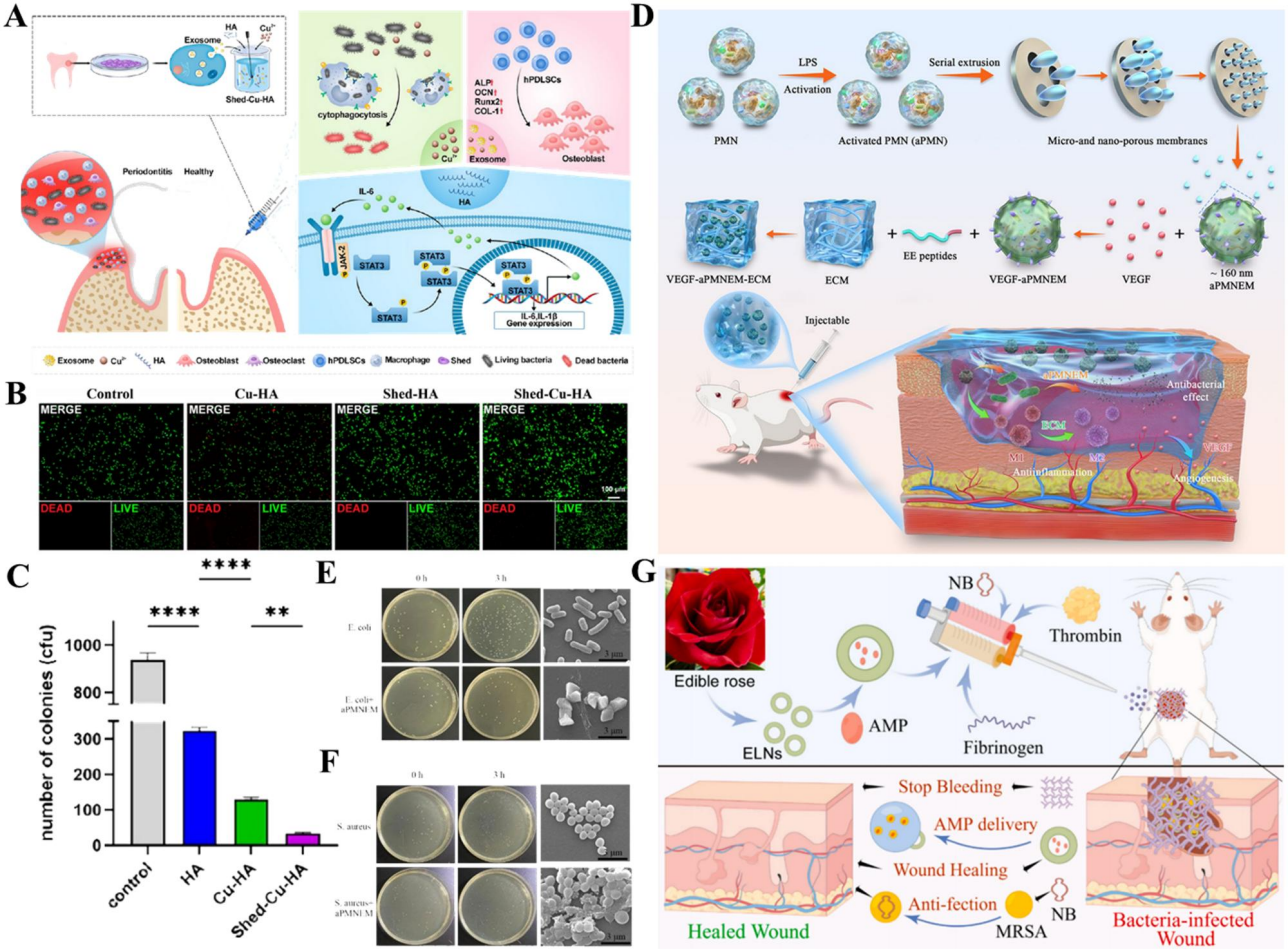
Exosome combined with hydrogel antibacterial cases (Note: In this figure, the symbol “*P<0.05, **P<0.015, ***P<0.0015, ****P<0.0001” indicates a highly significant statistical difference (p < 0.01) in quantitative comparisons between treated and control groups). (**A**) Schematic illustration of the synthesis of the Shed-Cu-HA hydrogel and its synergistic effects of Shed-exo, Cu^2+^, and an injectable HA hydrogel on antibacterial, anti-inflammatory and osteogenic activity for periodontal bone regeneration. (**B**) Fluorescent images of stained bacteria after treatment for Aa. (**C**) Antibacterial rate of the different hydrogels [144]. Copyright 2024, American Chemical Society. (**D**) Scheme showing the study procedures. A total of four main steps were performed. (**E**, **F**) aPMNEM inhibited the growth of *E. coli* and *S. aureus in vitro* [145]. Copyright 2023, BioMed Central Ltd. (**G**) Schematic illustration of the proposed pro-wound healing mechanism [71]. Copyright 2024, Elsevier B.V.

The integration of exosomes into hydrogels is particularly effective for managing chronic infections where biofilm persistence and immune dysregulation are prevalent. A prime example is the treatment of aggressive periodontitis caused by *Aggregatibacter actinomycetemcomitans* [143]. Addressing the dual challenge of bacterial evasion and tissue destruction, Yu *et al*. [144] engineered a hybrid system (Shed-Cu-HA) that incorporates stem cell-derived exosomes and copper ions within a hyaluronic acid (HA) matrix (Figure 13A). In this multifaceted design, the exosomes function as immunomodulators that enhance macrophage phagocytosis, while copper ions generate localized ROS to disrupt bacterial membranes. Crucially, the HA backbone not only ensures the controlled co-release of these agents but also physically impedes biofilm formation. This “search and destroy” strategy resulted in a 96.5% reduction in bacterial load *in vitro*, outperforming Cu-HA and HA alone, which achieved 86.1% and 65.4% antibacterial efficacy, respectively (Figure 13B and C). *In vivo*, Shed-Cu-HA was further validated in a mouse periodontitis model, where 2- and 4-week treatment reduced bacterial infection and inflammatory infiltration while promoting periodontal bone and collagen regeneration. Beyond acting as a passive depot, the HA hydrogel critically shaped this therapeutic effect through its optimized physicochemical properties. The selected 6 g/L HA formulation provided injectability for penetration into irregular periodontal pockets, sufficient mechanical stability and degradation resistance for local retention and a porous network for homogeneous loading and sustained co-release of Shed-exo and Cu^2+^. Thus, its superior antibacterial activity reflects not only the intrinsic bactericidal effect of Cu^2+^ and antiadhesive property of HA but also the hydrogel-mediated coordination of sustained ion release with exosome-driven immune regulation [144].

Similarly, in the context of diabetic wound healing complicated by neutrophil dysfunction, hydrogels can serve as bioactive dressings. Yu *et al*. [145] developed a thermosensitive hydrogel mimicking the extracellular matrix, loaded with neutrophil-derived exosome mimetics (aPMNEM). Enriched with potent antimicrobial proteins like myeloperoxidase and defensins, these vesicles delivered broad-spectrum bactericidal activity against *E. coli* and *S. aureus in vitro* (Figure 13D–F). Animal experiments further showed that aPMNEM inhibited bacterial growth in infected wounds and reduced local inflammatory cytokines, including TNF-α, IL-1β and IL-6, particularly at 48 h. Simultaneously, the hydrogel scaffold suppressed pro-inflammatory cytokines such as TNF-α, creating a permissive microenvironment for tissue regeneration. This demonstrates that hydrogel-exosome composites are not merely delivery systems but active modulators of the infection microenvironment. Here, thermosensitivity must be considered together with bulk degradation and viscoelasticity, because these parameters influence how long antimicrobial proteins remain concentrated within the wound and whether the matrix can maintain a moist yet structurally stable interface during the inflammatory phase.

Expanding the repertoire to plant-based systems, edible rose-derived ELNs offer a biocompatible and scalable alternative for targeting intracellular pathogens. Su *et al*. engineered these ELNs to encapsulate the antimicrobial peptide LL-37 and embedded them within a fibrin gel co-loaded with novobiocin. This composite system achieved a 2.5-fold increase in the clearance of intracellular MRSA compared to free peptides, leveraging the natural lipid composition of plant vesicles for enhanced cellular uptake (Figure 13G) [71]. The fibrin gel enabled 8-day sustained release of ELNs (AMP) and novobiocin while providing rapid hemostasis, reducing bleeding time from 87 s to 31 s in a mouse liver injury model. In an MRSA-infected skin defect model, ELNs (AMP)/NB-fibrin gel promoted near-complete wound closure by Day 14, increased granulation tissue and collagen deposition and reduced pro-inflammatory cytokines compared with PBS [142]. Mechanistically, the fibrin gel acted as more than a dressing: its *in situ* gelation allowed immediate wound coverage, while its porous and biodegradable network retained ELNs (AMP) and novobiocin locally, coordinating intracellular MRSA clearance by AMP with extracellular antibacterial activity from novobiocin [71]. While hydrogels excel in topical applications, they face limitations in penetrating deep tissue layers or biofilms. Microneedle technology addresses this by enabling the precise, minimally invasive delivery of therapeutics across the stratum corneum or mucosal barriers [146]. This approach is particularly advantageous for delivering high concentrations of exosomes directly to the infection nidus while minimizing systemic exposure. For microneedle systems, however, therapeutic success is inseparable from materials engineering. Mechanical strength determines whether the needles can reliably pierce necrotic or keratinized tissue, while dissolution rate controls how rapidly exosomes are deposited after insertion [147]. If the matrix is too soft, insertion efficiency decreases; if it is too brittle, premature fracture may occur, compromising both safety and delivery accuracy [148]. Compared with hydrogel dressings, microneedles therefore provide stronger evidence for enhanced tissue penetration, whereas hydrogels remain more suitable for broad surface coverage and prolonged topical retention.

**Figure 14:**
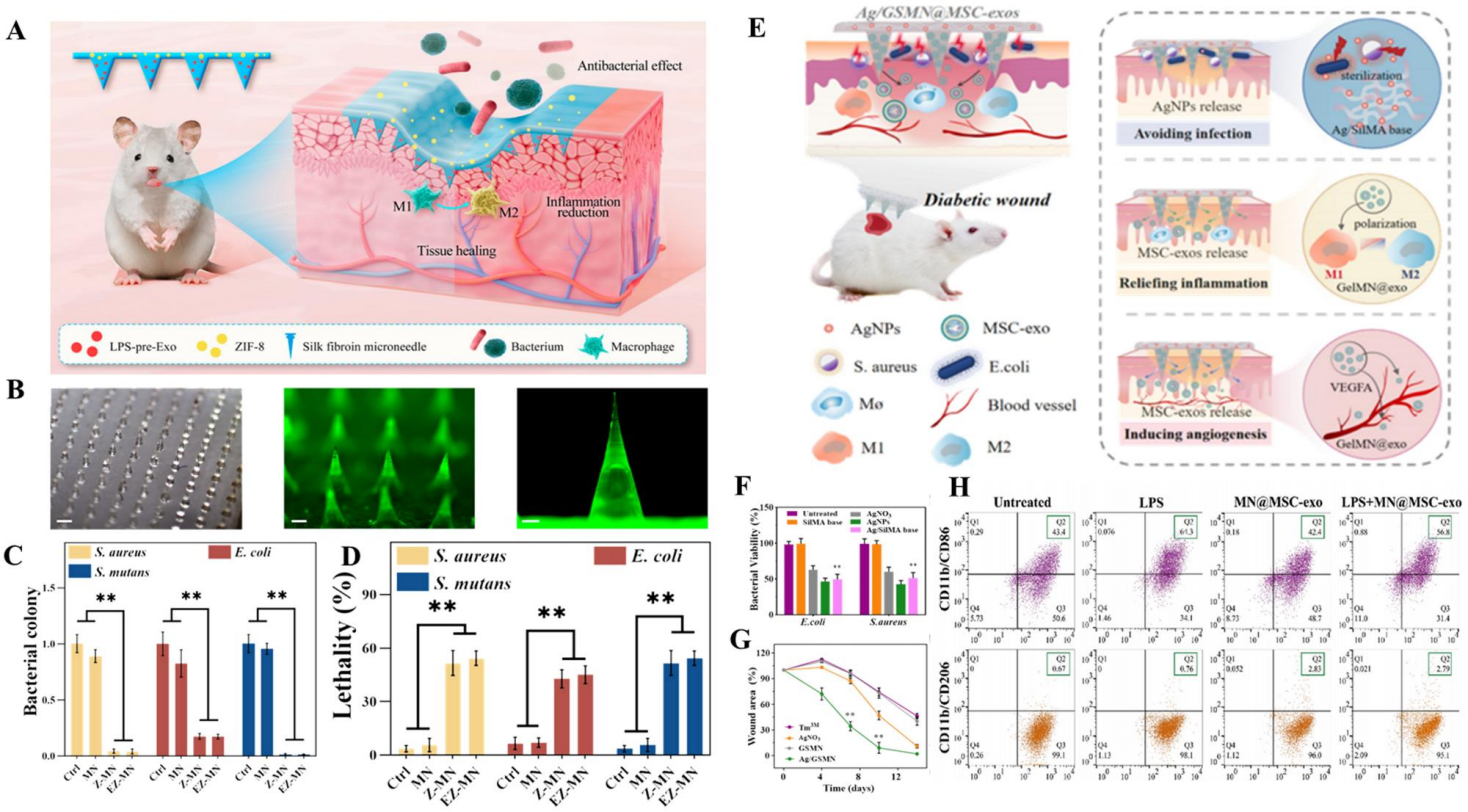
Exosomes combined with microneedles for antibacterial infection. (**A**) Schematic diagram of SF MNs for treatment of oral ulcers. (**B**) Characterization of SF MN and isolation of LPS-pre-Exos. (**C**) Quantitative analysis of bacterial colony. (**D**) Bacterial lethality rate. Scale bars: (1) 2 cm, (2) 200 μm. *P < 0.05, **P < 0.01 [79]. Copyright 2024, American Chemical Society. (**E**) Illustration of the MSC-exos loaded MN patch for promoting wound healing. (**F**) Bacterial viability with different treatments after 12 h incubation using the absorbance at 600 nm. (**G**) The quantitative results of wound area on the wound healing of *S. aureus* infection wound model. (**H**) Flow cytometry of macrophage polarization in RAW264.7 after stimulation. Macrophages were incubated with GSMN@MSC-exos in the presence of LPS (500 ng/mL) or the absence of LPS, PBS (untreated) and LPS for 24 h [149]. Copyright 2022, Elsevier Ltd.

In the challenging environment of the oral mucosa, adhesion and stability are paramount. To treat recurrent aphthous stomatitis, Ge *et al*. developed a dissolving microneedle patch (EZ-MN) based on silk fibroin, a material selected for its exceptional mechanical strength and wet adhesiveness (Figure 14A and B) [79]. This system employs a spatially resolved loading strategy: the microneedle tips contain exosomes derived from LPS-preconditioned MSCs, while the base is loaded with zinc-based metal-organic frameworks (ZIF-8). The preconditioning of exosomes amplifies their ability to shift macrophages from a pro-inflammatory M1 to a regenerative M2 phenotype via the NF-κB/NLRP3 pathway (Figure 14C and D) [79]. As the tips dissolve, these immunomodulatory vesicles are released deep into the ulcer bed to resolve inflammation, while the surface-bound ZIF-8 provides a sustained antimicrobial shield through zinc ion release. Notably, the high wet-state mechanical integrity of silk fibroin is particularly beneficial in the oral cavity, where saliva and tissue motion can otherwise weaken microneedle insertion and shorten residence time. In this study, the antibacterial and anti-inflammatory effects were first supported by *in vitro* assays, including sustained release of LPS-pre-Exos and Zn²^+^ for more than 7 days, inhibition of oral-related bacteria and reduced TNF-α and IL-6 levels in LPS-stimulated macrophages. Animal evidence was further provided in a rat oral ulcer model, where EZ-MN reduced mucosal TNF-α/IL-6 expression, increased M2 macrophage infiltration, enhanced collagen deposition and neovascularization and accelerated mucosal repair [79].

For diabetic skin wounds, mechanical robustness is essential for effective penetration. Gan *et al*. [149] engineered a hybrid microneedle patch featuring a methacrylated gelatin tip reinforced with silver nanoparticles and mesenchymal stem cell exosomes (Figure 14E–G). The exosomes released from the GelMA matrix facilitated vascular remodeling and immune homeostasis by fusing with endothelial cells and macrophages (Figure 14H). This sophisticated integration of nanomedicine and microneedle mechanics overcomes the barriers of necrotic tissue, ensuring that therapeutic agents reach viable cells to initiate repair. Mechanistically, this AgNPs/SilMA-GelMA microneedle patch couples early antibacterial protection with later regenerative delivery. The Ag/SilMA adhesive base inhibited both *E. coli* and *S. aureus*, maintaining antibacterial rates above 50% even at low Ag/SilMA concentrations, and in *S. aureus*-infected diabetic rat wounds, it markedly reduced bacterial colonies on Days 7 and 10. Meanwhile, the GelMA microneedle tips provided sufficient stiffness and fracture resistance for reproducible insertion into mechanically heterogeneous diabetic wounds, limiting exosome loss and enabling local sustained MSC-exosome release. These MSC-exosomes were internalized by endothelial cells, promoted migration and tube formation and shifted macrophages toward a reparative phenotype, with M1 macrophages decreasing from 64.12 ± 2.67% to 56.71 ± 5.80% and M2 macrophages increasing from 0.73 ± 0.12% to 2.81 ± 0.35%. *In vivo*, this spatiotemporal design reduced IL-6 expression by Day 14, enhanced CD31-positive angiogenesis and achieved nearly complete wound closure by Day 13 [149].

The convergence of exosome biology with advanced biomaterials represents a paradigm shift in infection management. Hydrogels provide the necessary spatiotemporal control for sustained release, transforming exosomes from transient signaling molecules into long-acting therapeutic depots [150]. Microneedles further refine this by offering deep tissue penetration and precise targeting [151]. More broadly, rational tuning of pore architecture, degradation behavior and mechanical performance converts biomaterials from passive carriers into active regulators of exosome biodistribution and antibacterial efficacy.

However, persistent challenges such as the limited payload capacity of microneedles and the difficulty of controlling release kinetics in dynamic infection environments remain. To address these, the field is moving toward stimuli-responsive systems, such as incorporating photothermal agents. These smart platforms utilize near-infrared (NIR) light to trigger on-demand exosome release and generate localized hyperthermia, physically disrupting biofilms and synergizing with the biological effects of exosomes to eradicate recalcitrant infections [152]. This evolution from passive carriers to active, responsive systems holds the promise of achieving total eradication of multidrug-resistant pathogens. Thus, photothermal-exosome platforms may offer the strongest theoretical advantage for biofilm disruption, but their evidence level should be distinguished from microneedle systems that have already been validated in infected or inflammatory animal wound models.

### Exosomes combined with phototherapy

Managing chronic wounds, particularly in diabetic patients, is often hindered by the dual challenge of multidrug-resistant bacterial infections and a persistently inflamed microenvironment. Conventional antibiotic therapies are frequently ineffective against biofilms and may even exacerbate immune dysregulation. In recent years, the integration of exosome-based therapeutics with photothermal therapy (PTT) has emerged as a promising synergistic strategy [153]. Exosomes function as potent nanocarriers for immunomodulation and tissue regeneration, while photothermal agents offer a physical, resistance-free mechanism for bacterial elimination. Together, this dual-modal approach not only addresses the immediate threat of infection but also fosters a favorable microenvironment conducive to subsequent tissue repair. Compared with single exosome delivery or conventional hydrogel dressings, exosome-photothermal systems show clearer advantages in early antibacterial efficacy and infection control, whereas exosomes mainly contribute to later-stage inflammation resolution, angiogenesis, collagen deposition and epithelial repair [154].

**Figure 15:**
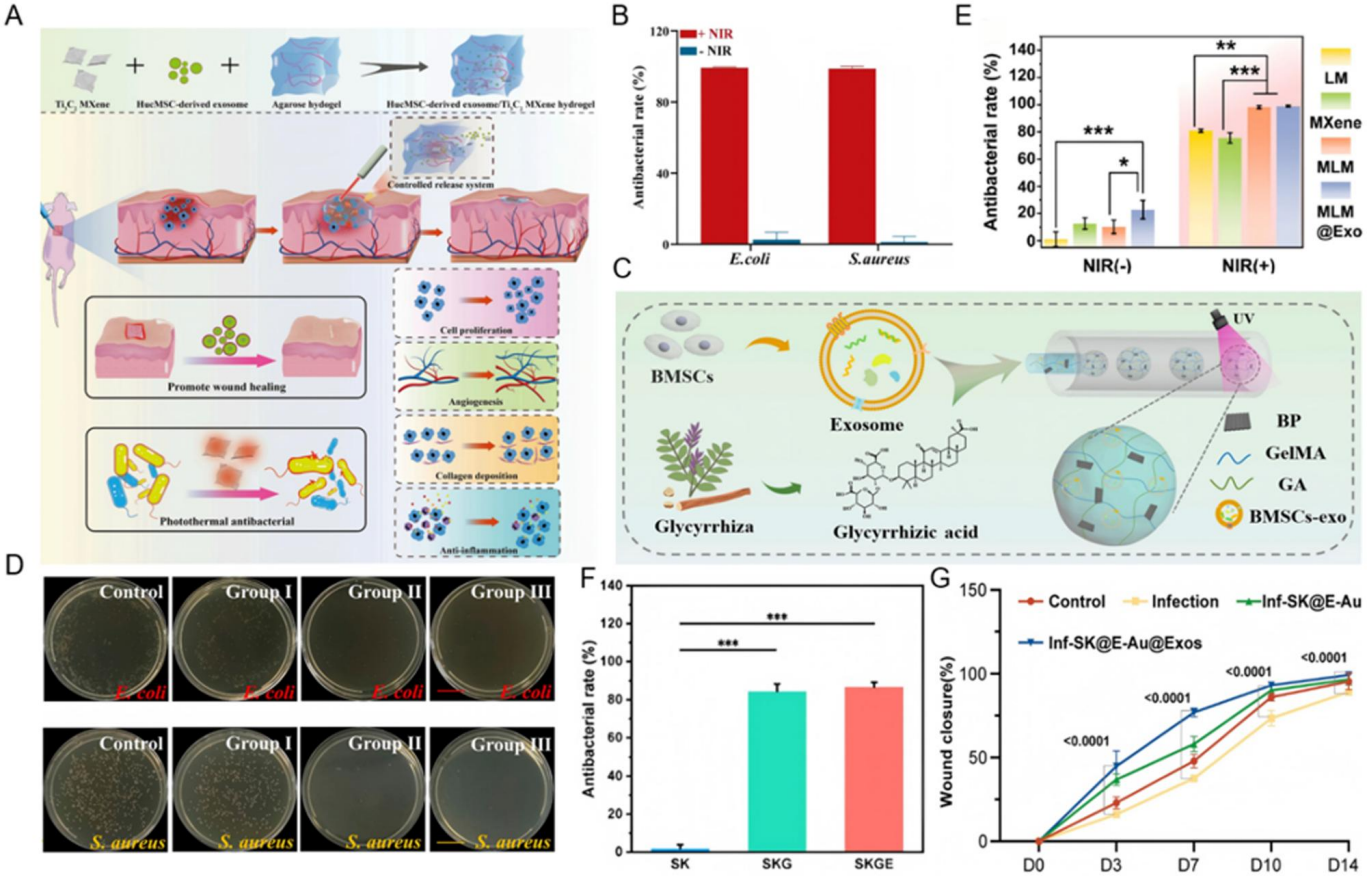
Combined antibacterial effect of exosomes and photothermal system (Note: In this figure, the symbol “*P<0.05, **P<0.015, ***P<0.0015, ****P<0.0001” indicates a highly significant statistical difference in quantitative comparisons between treated and control groups). (**A**) Schematic diagram of the antibacterial ability of MLM bio-HJs under irradiation stimulation *in vitro*. (**B**) Antibacterial ratio of hucMSC-derived exosome/Ti3C2 MXene hydrogel to *E. coli* and *S. aureus* with and without NIR laser irradiation. Error bars were defined as the standard deviation [155]. Copyright 2024, Elsevier B.V. (**C**) Schematic diagram of microfluidic preparation of exosome-encapsulated Chinese herb hydrogel microparticles. The healing process of the diabetic wound accelerated by hydrogel microparticles with exosomes and Chinese herb. (**D**) Images of bacterial colonies on culture plates of different groups [156]. Copyright 2024, Luting Zhang et al. (**E**) Antibacterial rates of *S. aureus* based on bacterial plate coating [153]. Copyright 2023, American Chemical Society. (**F**) antimicrobial rate statistics. (**G**) Wound healing rate [157]. Copyright 2025, Elsevier Ltd.

A major limitation of exosome therapy is the rapid clearance and short half-life of exosomes *in vivo*. To overcome this, recent studies have developed smart hydrogel systems that leverage the photothermal effect for on-demand exosome release. For instance, Teng *et al*. [155] designed an agarose-based hydrogel incorporating Ti_3_C_2_ MXene and human umbilical cord mesenchymal stem cell-derived exosomes (hucMSC-Exos) (Figure 15A). Upon NIR irradiation, MXene-mediated photothermal conversion induces a phase transition in the agarose matrix, enabling precise spatiotemporal control over exosome release (Figure 15B). This system achieved high *in vitro* antibacterial rates against *E. coli* and *S. aureus*, approximately 99.5% and 98.9%, respectively, and promoted HaCaT cell migration, with a scratch closure ratio of about 74.8%, indicating that its evidence includes both antibacterial and pro-regenerative cellular assays. In a complementary approach, Zhang *et al*. [156] incorporated black phosphorus (BP) into glycyrrhizic acid (GA) hydrogel microparticles, achieving NIR-triggered exosome release via a reversible gel–sol phase transition of GA (Figure 15C and D). This platform combined antibacterial GA, photothermal BP and BMSC-derived exosomes; *in vitro*, GA-containing microparticles showed nearly complete bacterial killing, while in a bacteria-infected diabetic wound model, the NIR-treated exosome-loaded microparticles reached about 87% wound closure by Day 11, supporting both *in vitro* and animal-level efficacy. Beyond controlled release, the photothermal effect itself provides robust antibacterial activity. Li *et al*. [153] demonstrated that gold nanorods (AuNRs) embedded in an antiswelling hydrogel could generate localized heating under NIR irradiation, effectively eliminating bacterial infections. Simultaneously, co-delivered M2-Exos reduced oxidative stress and suppressed inflammation (Figure 15E). This spatiotemporally coordinated strategy ensures that the wound bed is sterilized prior to the peak activity of regenerative exosomes. Here, the antiswelling property is also mechanically meaningful, because it helps the hydrogel retain dimensional stability during repeated fluid uptake and laser exposure, thereby preserving uniform heat distribution and consistent exosome release behavior. In addition, exosome-decorated MXene/liquid-metal bio-heterojunctions have shown that reducing the transfer distance of heat and ROS can enhance photothermal/photodynamic bacterial killing and biofilm disruption, although this evidence is mainly derived from *in vitro* bacterial and biofilm models plus general infected wound models rather than diabetic chronic wound models.

Beyond their direct antimicrobial effects, these exosome-photothermal platforms actively engage in remodeling the immune microenvironment. Persistent inflammation, typically marked by M1 macrophage polarization, is a hallmark of chronic wounds. Emerging evidence suggests that such platforms can effectively shift macrophage polarization from the pro-inflammatory M1 phenotype toward the anti-inflammatory, pro-regenerative M2 phenotype. For example, Duan *et al*. developed a silk protein hydrogel encapsulating broccoli-derived exosomes (Bro-Exos) and EGCG-gold nanoparticles (Figure 15F) [157]. This system achieved near-complete MRSA eradication via photothermal heating and significantly downregulated NF-κB signaling, thereby promoting macrophage transition to a reparative state (Figure 15G). In a mouse MRSA-infected wound model, SK@E-Au@Exos accelerated wound closure to approximately 76.9% on Day 7 and 99.3% on Day 14, while reducing inflammatory infiltration, epidermal hyperplasia and scar index. These findings indicate that its immunomodulatory conclusions are supported not only by *in vitro* macrophage and ROS assays, but also by animal histology and wound-healing outcomes. Similarly, Li *et al*. integrated silver-decorated polydopamine nanozymes with MSC-derived exosomes within an HA-recombinant human collagen scaffold [158]. The Ag-PDA nanozymes not only provided photothermal antibacterial activity but also scavenged excessive ROS (ROS), while MSC-Exos promoted angiogenesis and suppressed pro-inflammatory cytokines such as TNF-α and IL-6. These synergistic effects highlight the dual role of photothermal agents in pathogen clearance and ROS mitigation, complemented by exosomes that supply the regenerative cues essential for vascularization and scarless wound healing.

Collectively, these studies underscore the promise of combining exosomes with photothermal and catalytic materials to engineer multifunctional wound dressings. In such systems, the photothermal component acts as both a “defense shield” against pathogens and a “trigger” for on-demand exosome release, while exosomes serve as a “regenerative engine” driving immunomodulation and tissue repair.

Beyond photothermal strategies, nanozyme-based catalytic materials, such as CuCo_2_O_4_ and related peroxidase-/oxidase-like nanozymes, offer another promising route for antibacterial therapy by catalyzing reactive oxygen species generation, disrupting bacterial membranes and remodeling the infected wound microenvironment [159]. In particular, Cu-based nanomaterials can release Cu ions and induce oxidative stress, membrane damage and metabolic dysfunction, thereby promoting bacterial death [160, 161]. Future research should focus on synergistic antibacterial strategies integrating photothermal functional materials with exosomes. Photothermal/catalytic platforms can enable rapid bacterial eradication and biofilm disruption, while exosomes may modulate inflammation, promote angiogenesis and support tissue regeneration. Further studies should strengthen *in vivo* mechanistic evidence, optimize photothermal wavelength and tissue penetration and clarify how different exosome sources regulate immune responses under photothermal antibacterial conditions (Table 2).

**Table 2:** Exosome-based multi-antibacterial strategy.

Strategy	Source	Effector	Target	Core mechanism	Ref.
Direct antibacterial effect	Peppermint	Endogenous components	*Micrococcus luteus*, *Escherichia coli*	Disruption of bacterial cell membrane integrity, leading to leakage of contents.	[92]
	Honeybee	Endogenous AMP MRJP1	*Staphylococcus aureus*	Disruption of physical structure, eradicating pathogen defense matrix.	[96]
	Bovine colostrum	Biophysical disruption	*S. aureus*	Blockade of core energy metabolism pathways, disrupting bacterial cell membrane integrity.	[83]
	*Arabidopsis thaliana*	Endogenous siRNA	*Botrytis cinerea*	Targeted silencing of pathogen-specific virulence genes.	[102]
	Mammalian cells	Endogenous AGO2	MRSA	Genomic silencing of resistance genes, restoring antibiotic susceptibility.	[103]
	Human airway epithelial cells	Endogenous miRNA let-7b-5p	*Pseudomonas aeruginosa*	Disruption of bacterial biofilms, reversing antibiotic resistance.	[80]
	*Taraxacum mongolicum*	Self as decoy receptor	*S. aureus*	Acting as biomimetic receptors to competitively bind and neutralize exotoxins, protecting host cells.	[109]
	Ginger	Self as decoy receptor	*S. aureus* & its constructs	Acting as biomimetic ‘nanosponges’ to bind and neutralize bacterial toxins.	[162]
Immune regulation function	hUCMSCs	Endogenous immunomodulatory miRNAs & proteins	MRSA	Strong induction of macrophage polarization toward a pro-inflammatory (M1) phenotype to enhance bactericidal activity.	[163]
	M1-macrophages	Endogenous pro-inflammatory cytokines, specific miRNAs	Deep tissue infections & intracellular pathogens	Chemotaxis of peripheral blood neutrophils to the local infection site, enhancing phagocytosis.	[113]
	Macrophages	SCIMP protein	*E. coli*	Specific chemotaxis and recruitment of immune cells (e.g. neutrophils) to the site of infection.	[120]
	M2-Macrophages	Endogenous anti-inflammatory miRNAs & proteins	*Porphyromonas gingivalis*	Mediating immune microenvironment remodeling, suppressing cytokine storms and promoting tissue repair.	[124]
Drug nano-carrier	Macrophages	Linezolid	*S. aureus*	Mediating membrane fusion for precise intracellular or intra-bacterial delivery of antibiotics.	[164]
	Bovine milk	α-mangostin	*E. coli*	Penetration and disruption of bacterial biofilms, enhancing local drug concentration.	[165]
	Bovine milk	Polymyxin B; Isobavachalcone	MDR bacterial pathogens	Breaking the lysosomal barrier for highly efficient intracellular delivery and bactericidal action.	[128]
	Garlic	Vancomycin	*S. aureus*	Targeted endocytosis into infected cells, breaking physical bacterial barriers.	[70]
	Bacteria	Lysozyme	Antibiotics (broad)	Biomimetic camouflage utilizing natural membrane structures to evade host immune clearance and prolong half-life.	[166]
	Macrophages	Lysostaphin	Intracellular MRSA	Surface receptor modification for pathogen-specific recognition and targeted intracellular delivery.	[131]
	Macrophages	AMPs	MRSA, *E. coli*	Engineered exosome surfaces for prolonged blood circulation and enhanced targeting.	[132]
Engineering-targeted modification	Fresh seaweed	Aptamer	*E. coli* & *S. aureus*	Surface conjugation of specific aptamers for high-affinity recognition and binding of pathogens.	[167]
	*S. aureus*	Mesoporous silica nanoparticles (MSN)	*S. aureus*	Biomimetic delivery system utilizing homologous targeting to enhance bactericide accumulation at infection sites.	[168]
	Mammalian cells	High-density coating/covalently linked cationic AMPs	Sepsis-associated circulating resistant bacteria	High-density AMP modification for rapid eradication of circulating pathogens and control of endotoxin release.	[169]
Joint functional materials	hUCMSCs	HA hydrogel	MRSA-infected diabetic wounds	Hydrogel-mediated spatiotemporal sustained release, synergizing with exosomal immunomodulation to promote wound healing.	[163]
	MSCs	AgNPs + quaternized chitosan hydrogel + calcium alginate microspheres	*P. aeruginosa*, wound infections	Synergy between nanosilver physical sterilization and exosome-induced angiogenesis for accelerated tissue repair.	[170]
	SHEDs	Cu2+, HA hydrogel	*Aggregatibacter actinomycetemcomitans*	Synergy between Cu2+-mediated in situ physical sterilization and exosome-mediated periodontal immunomodulation.	[144]
	LPS-stimulated cells	Endogenous MPO, dECM hydrogel	*S. aureus*, *E. coli*	In situ antibiotic-free sterilization, synergistically inducing M1 macrophage polarization for anti-infection effects.	[145]
	Edible rose	AMP/novobiocin sodium + fibrin gel	MRSA	Gel barrier penetration for spatiotemporal responsive release of antibiotics and exosomes.	[71]
	LPS-stimulated cells	ZIF-8 (Zn2+), silk fibroin	Secondary infections in oral ulcers	Microneedle system penetrating physical barriers, combining Zn2+ sustained release inhibition with exosomal synergy.	[79]
	MSCs	Silver nanoparticles, silk fibroin	*S. aureus*, *E. coli*	Physical substrate adhesion and sustained Ag+ release for long-term synergistic defense.	[149]
	Mammalian cells	MXene & liquid metal (LM)	Deep-seated MRSA infections	Deep tissue penetration, synergizing near-infrared (NIR) photothermal sterilization with exosome sustained release.	
	hUCMSCs	2D transition metal carbide (Ti3C2 MXene)	Common pathogens in skin wound infections	NIR-triggered photothermal effect for direct pathogen eradication while stimulating exosome release for angiogenesis.	
	*Lactobacillus bulgaricus*	PCPH	Mixed infection flora in chronic wounds	Alleviating local hypoxia microenvironment and resolving chronic inflammation for durable sterilization.	[171]
	BMSCs	GA, BP nanosheets	Mixed infection flora in chronic wounds	NIR-triggered photothermal sterilization with smart regulation of exosome release, predominantly driving angiogenesis.	[156]
	M2-macrophages	AuNRs	Pathogens in diabetic defects/oral mucosal ulcers	AuNRs-mediated photothermal sterilization, scavenging local ROS to improve the oxidative stress microenvironment.	[153]

## Exosome preclinical application at infection sites

### Diagnosis and monitoring of bacterial infections

Real-time monitoring of disease dynamics offers substantial advantages by enabling the timely detection of infection onset, progression and dissemination, thereby informing rapid clinical responses and optimizing resource allocation [172]. Accurate monitoring supports a precise assessment of disease spread and severity, yielding critical data for both epidemiological research and targeted therapeutic interventions while enhancing public health preparedness. Meeting this demand, however, requires overcoming the limitations of traditional bacterial diagnostic methods. Conventional approaches such as culture and biochemical identification, though specific, often lack the speed required for urgent clinical decision-making [173]. Consequently, the identification of novel, rapid and sensitive biomarkers is paramount for improving the early diagnosis and management of bacterial infections.

This imperative has driven significant interest in exosomes as a rich source of molecular biomarkers. These extracellular vesicles carry diverse bioactive molecules—including proteins, lipids and nucleic acids—and play a key role in intercellular communication within the extracellular microenvironment [174]. Their advantages over other liquid biopsy components, such as enhanced stability and the protection of cargo from degradation, position exosomes as compelling candidates for clinical biomarker development [175]. Released into biofluids through regulated secretory pathways, exosomes facilitate vital material transfer and signaling between cells, reflecting the physiological or pathological state of their parental source. These unique biological properties, combined with their molecular richness, have established exosomes as a promising medium for biomarker discovery across various medical fields.

Driven by this clinical potential, the past decade has witnessed substantial advances in ultrasensitive exosome detection technologies [176]. Innovative biosensing platforms employing optical, electrochemical and electrical principles have enabled significant progress in high-throughput isolation, rapid screening and sophisticated analytical profiling of exosomes [177]. Such technological developments are crucial for translating exosome-based diagnostics into practical clinical tools.

Real-time monitoring of disease dynamics is critical for capturing infection onset and progression, yet traditional diagnostic approaches, such as culture and biochemical identification, frequently lack the speed required for urgent clinical decision-making [172, 173]. Consequently, exosomes have attracted significant interest as a rich source of liquid biopsy biomarkers due to their diverse bioactive cargo and superior stability in biofluids, which dynamically reflect the pathological state of their parental cells [174, 175]. To facilitate their clinical translation, the past decade has witnessed substantial progress in ultrasensitive biosensing platforms—including optical, electrochemical and electrical systems—enabling high-throughput isolation and rapid analytical profiling of exosomes [176, 177].

The practical application of exosomes as infection biomarkers is well illustrated in tuberculosis (TB) research. While conventional diagnostics like sputum smear microscopy and molecular assays face sensitivity and operational constraints in resource-limited settings, *M. tuberculosis* (MTB) infection significantly alters the concentration and molecular composition of circulating host exosomes [178]. These vesicles encapsulate specific miRNAs, protecting them from ribonuclease degradation and conferring remarkable stability in blood and other biofluids [179]. This infection-specific alteration in miRNA profiles establishes exosome-associated signatures as highly promising candidate biomarkers for early diagnosis and monitoring.

However, fully translating exosome-based diagnostics into routine clinical practice for bacterial infections remains an evolving frontier that requires overcoming several critical translational barriers.

First, the lack of standardization in isolation and purification methodologies presents a primary hurdle; current techniques often suffer from low purity or low throughput, and specifically in infectious diseases, differentiating host-derived exosomes from co-existing bacterial extracellular vesicles in complex biological matrices remains technically challenging. Second, substantial biological heterogeneity and confounding clinical variables can severely compromise diagnostic specificity, as exosomal cargo profiles are highly sensitive to patient age, comorbidities and nonspecific systemic inflammatory responses. Third, limited technological scalability for point-of-care (POC) applications restricts widespread adoption, given that most advanced ultrasensitive biosensing platforms still rely on high-cost laboratory instrumentation and specialized personnel rather than rapid, user-friendly and cost-effective formats suitable for urgent clinical environments. Fourth, the deficit in large-scale prospective clinical validation and standardized reference controls creates a bottleneck, hindering the reproducibility of diagnostic accuracy and subsequent regulatory approval.

Addressing these intertwined challenges through interdisciplinary advancements in separation science, sensor technology and bioinformatics will be essential to realize robust, clinically viable platforms for real-time infection monitoring based on exosomal biomarkers.

### Treatment of skin and soft tissue infections

The escalating prevalence of antibiotic-resistant pathogens, including MRSA and *P. aeruginosa*, highlights the critical limitations of conventional therapies for chronic skin wounds [180]. These bacteria frequently establish resilient biofilms, evade host immune defenses and perpetuate destructive inflammatory cycles, resulting in impaired healing and systemic complications. In response to this challenge, exosomes—nanoscale extracellular vesicles derived from stem cells—have emerged as promising multifunctional agents uniquely capable of concurrently targeting persistent microbial threats and dysregulated tissue repair processes [181].

Recent advances demonstrate that integrating exosomes with sophisticated biomaterial delivery systems substantially enhances their therapeutic potential [182]. Illustrating this synergy, Qian *et al*. engineered an asymmetric chitosan-silk fibroin dressing incorporating silver nanoparticle-adsorbed exosomes to combat multidrug-resistant *P. aeruginosa*. Within this composite system, the silver nanoparticles delivered rapid bactericidal effects through membrane disruption, while hucMSC-Exos promoted critical fibroblast proliferation and angiogenesis via VEGF and FGF signaling pathways. This dual-action strategy achieved a remarkable reduction in bacterial load *in vitro* and accelerated wound closure in murine models, significantly outperforming mono-therapies [182]. Building upon the concept of intelligent delivery, Wang *et al*. developed a pH-responsive polysaccharide hydrogel loaded with adipose-derived stromal cell exosomes. This system enabled sustained exosome release specifically within the acidic microenvironment of diabetic wounds, effectively eradicating MRSA biofilms while enhancing endothelial cell migration and tube formation through the upregulation of the pro-angiogenic microRNA miR-126 [183]. Collectively, these studies illustrate how tailored biomaterials overcome inherent limitations of exosome therapy, such as rapid clearance and enzymatic degradation, enabling localized and responsive delivery aligned with infection dynamics.

Beyond direct antimicrobial and pro-regenerative activities, exosomes further demonstrate a pivotal capacity to resolve the chronic inflammation that sustains persistent infections. Chronic wounds typically exhibit a sustained pro-inflammatory state dominated by M1 macrophages, creating a significant barrier to healing [174]. Complementing antimicrobial strategies, Yue *et al*. targeted this inflammatory imbalance by embedding hucMSC-Exos into a carboxymethyl chitosan/oxidized HA hydrogel. This approach effectively polarized macrophages toward the regenerative, anti-inflammatory M2 phenotype [184]. In *S. aureus*-infected diabetic mouse models, the hydrogel formulation reduced detrimental TNF-α levels while significantly boosting secretion of reparative cytokines such as IL-10 and TGF-β. Consequently, enhanced collagen deposition and robust re-epithelialization ensued, culminating in markedly improved wound closure rates compared to untreated controls. This strategy powerfully underscores the capacity of exosomes, when strategically delivered, to reprogram detrimental immune responses and synergize with biomaterials to restore essential tissue homeostasis [184].

In conclusion, exosome-based therapies represent a paradigm shift in managing complex bacterial skin infections by integrating potent antimicrobial action, sophisticated immunomodulation and pro-regenerative functions within a single platform. Their inherent compatibility with advanced biomaterials, which facilitate sustained release, microenvironmental responsiveness and enhanced biofilm penetration, directly addresses the multifactorial pathology of chronic, resistant infections [185]. As antibiotic resistance escalates relentlessly, these innovative combinatorial approaches offer a compelling blueprint for next-generation wound care, effectively bridging the critical gap between stringent infection control and comprehensive functional tissue regeneration. Future research should prioritize optimizing exosome cargo engineering and biomaterial design parameters to tailor therapies precisely for specific pathogens and diverse clinical populations.

### Treatment of bone infections

The complex pathology of osteomyelitis demands integrated therapeutic strategies capable of simultaneously addressing persistent infection, dysregulated inflammation and compromised bone regeneration [186]. Exosome-based platforms are uniquely positioned to meet this multifaceted challenge by harnessing the innate biological cargo of extracellular vesicles to orchestrate coordinated therapeutic responses [187]. A compelling demonstration of this integrated approach is the RAB-EXO system, engineered by Chen *et al*. [103] to actively target the infected bone niche. These macrophage-derived nanovesicles incorporate a triblock peptide featuring a bone-homing domain, a ROS-responsive cleavable linker and an antimicrobial peptide domain. Within the inflamed osteomyelitic microenvironment characterized by elevated ROS, the linker degrades to trigger localized release of LL-37 analog AMPs, directly eradicating pathogens such as MRSA. In a rat model of established MRSA osteomyelitis, this targeted delivery mechanism achieved a substantial reduction in bacterial burden while concurrently facilitating significant bone recovery, restoring bone volume fraction by approximately 40%. This dual outcome was attributed to the system’s ability to modulate host immune responses while stimulating osteoblast activity [132].

Building upon this foundation of exosome-mediated immunomodulation, further research has elucidated mechanisms to actively reprogram the dysregulated immune landscape in osteomyelitis. Pan *et al*. demonstrated that exosomes engineered to deliver the anti-inflammatory cytokine IL-10 and the microRNA miR-146a could effectively repolarize pro-inflammatory M1 macrophages toward a reparative M2 phenotype [188]. This phenotypic shift is pivotal, as it attenuates persistent inflammation, a major barrier to healing, while concurrently enhancing the tissue repair processes essential for functional bone recovery.

Complementing these immunological benefits, mesenchymal stem cell-derived exosomes exhibit profound intrinsic osteogenic potential, offering a critical dimension for bone regeneration [189]. These vesicles function as natural carriers of potent osteoinductive molecules, including specific miRNAs such as miR-136-5p and key growth factors like bone morphogenetic protein-2 [190]. Their cargo activates fundamental osteogenic pathways, including Wnt/β-catenin and SMAD/RUNX2 signaling, to robustly stimulate osteoblast differentiation and bone matrix mineralization. This regenerative capacity was clearly demonstrated in a preclinical model where mesenchymal stem cell exosomes accelerated the repair of *P. aeruginosa*-infected calvarial defects, significantly elevating alkaline phosphatase activity, upregulating type I collagen expression and achieving complete bridging of critical-sized bone defects within 28 days. Beyond promoting bone formation, these exosomes also demonstrate a protective role against bacterial virulence factors, such as by neutralizing *S. aureus* protein A-mediated osteoblast apoptosis through suppression of caspase-3 activation and upregulation of the anti-apoptotic protein Bcl-2 [191].

Collectively, these advances underscore the transformative potential of exosome-based strategies. By integrating targeted antimicrobial delivery, intelligent immunomodulation and potent osteogenic signaling within a single biological nanoparticle platform, engineered exosomes offer a sophisticated and integrated therapeutic solution tailored to address the multifactorial pathology of osteomyelitis.

### Treatment of pulmonary infections

Bacterial pneumonia remains a significant global health burden, compounded by escalating antibiotic resistance and the inherent limitations of conventional drug delivery systems [192]. Within this challenging therapeutic landscape, exosomes have emerged as highly promising nanoscale vehicles, leveraging their natural biocompatibility and intrinsic capacity to traverse biological barriers for targeted intervention in the infected lung [193]. This potential is particularly critical against major etiological agents such as *P. aeruginosa* and MRSA, pathogens noted for their immune evasion and resistance mechanisms, which frequently cause difficult-to-treat hospital-acquired pneumonia [194]. Engineered exosomes offer a compelling dual advantage by enabling the precise delivery of antimicrobial agents while simultaneously modulating detrimental host immune responses [195].

A principal strategy to harness this potential involves modifying exosome surfaces to enhance pulmonary accumulation. Qiu *et al*. [195] exemplified this approach by engineering exosomes to display an ischemic myocardial targeting peptide fused to the Lamp-2b protein, conferring specific affinity for integrin receptors enriched in lung tissue. This active targeting mechanism significantly increased exosome retention in murine models of lung injury, illustrating a method to concentrate therapeutic payloads at the infection site while minimizing systemic exposure and off-target effects.

Beyond spatial targeting, exosomes function as sophisticated immunomodulators to resolve infection and mitigate tissue damage. Wang *et al*. [196] demonstrated that curcumin-loaded extracellular vesicles reduced pro-inflammatory cytokines and enhanced bacterial clearance in mice infected with *Actinobacillus pleuropneumoniae*, showcasing combined anti-inflammatory and antimicrobial effects. Further elucidating specific mechanisms, Jia *et al*. [118] revealed that NK cell-derived exosomes bolster host defenses against *P. aeruginosa* while reducing associated lung pathology. In *Klebsiella pneumoniae* infection, exosomes from tracheal epithelial cells deliver miR-21-5p to alveolar macrophages, where it silences the PIK3CD gene and induces autophagy. This process reduces pyroptosis and ROS production, alleviating the cytokine storm characteristic of acute lung injury and enhancing bacterial clearance—a mechanism also validated in models of MRSA pneumonia [132, 167]. These findings highlight the capacity of exosomes to finely balance effective pathogen clearance with necessary tissue protection.

In summary, exosome-based therapies represent a transformative paradigm for combating bacterial pneumonia, integrating targeted delivery with intelligent immune reprogramming within a single platform. Preclinical successes, including the resolution of MRSA-driven pulmonary inflammation by mesenchymal stem cell exosomes, strongly support their translational potential (Table 3).

**Table 3:** Summary of exosome-based strategies for infected tissue repair.

Application	Exosome source	Delivery/material strategy	Infection model	Route	Main outcomes	Key limitations
Infected skin wound	HUMSC-Exos	Chitosan-silk fibroin dressing loaded with AgNP-Exos	*P. aeruginosa*-infected full-thickness mouse wound	Topical dressing	Faster closure, lower bacterial burden, enhanced collagen deposition, angiogenesis and nerve repair	Short-term mouse model; long-term Ag safety and exosome standardization unclear [180]
Chronic inflammatory skin wound	HUCMSC-Exos	CMCS/OHA hydrogel	*S. aureus*-infected mouse inflammatory wound	Topical hydrogel	Sustained Exo release, M2 macrophage polarization, reduced TNF-α/IL-6, improved re-epithelialization and collagen deposition	Limited direct antibacterial activity; no biofilm, diabetic or large-animal model [183]
Diabetic skin wound	ADSC-Exos	Injectable FEP hydrogel	STZ-induced diabetic mouse wound; *in vitro* antibacterial tests	Local hydrogel application	Sustained release, improved angiogenesis, granulation, collagen remodeling and appendage regeneration	Not a true infected diabetic wound model; antibacterial evidence mainly *in vitro* [184]
Osteomyelitis	M2 macrophage-Exos	RAB peptide-engineered Exos via click chemistry	MRSA rat osteomyelitis model	Intravenous injection	Reduced bacterial load and inflammation, promoted M2 polarization and bone repair within 28 days	Small-animal model; limited biofilm/implant infection evidence; engineering safety unresolved [103]
Osteomyelitis mechanism	BMSC-Exos	Free Exos	SPA-treated MC3T3-E1 *in vitro* model	*In vitro* co-culture	Enhanced osteoblast proliferation, osteogenesis and autophagy; reduced TNF-α/IL-1β/IL-6	No *in vivo* infection model, delivery system, pharmacokinetics or safety data [188]
Bone repair platform	Various Exos, mainly MSC-Exos	Nano-/injectable hydrogel-Exo systems	Review evidence across bone defects and orthopedic diseases	Mostly local injection	Improved Exo retention and sustained release for bone repair	Infection-specific evidence is limited; standardization and material safety remain unclear [188]
Bacterial pneumonia	PTEC-Exos carrying miR-21-5p	Natural Exos or miR-21-5p delivery	Mouse pneumonia induced by APP; validation with *K. pneumoniae* and MRSA	Intravenous pretreatment	Improved survival, reduced lung bacterial load, cytokines and macrophage pyroptosis	Mainly prophylactic; short follow-up; lung targeting and safety need validation [195]
*P. aeruginosa* lung infection	NK cell-Exos	Natural NK-Exos	Mouse *P. aeruginosa* lung infection	Intravenous injection	Promoted M1 macrophage polarization, reduced bacterial burden and lung injury	Stage-dependent inflammatory risk; limited dose-response, biodistribution and safety data [196]
Lung-targeted delivery	Mainly MSC-Exos and engineered Exos	Peptide-modified Exos, e.g. RGD, GE11, tLyP-1, CP05, Lamp-2b fusion	Review evidence across ALI/ARDS, COVID-19, lung cancer and fibrosis	Inhalation, local or systemic delivery	May improve lung targeting and reduce liver/spleen clearance	Few clinical data; preparation, peptide selection and engineering safety need standardization [118]

## Conclusion and prospect

In summary, exosomes and prokaryotic extracellular vesicles have emerged as pivotal, dualistic orchestrators of the biological arms race during bacterial pathogenesis, displaying highly specialized regulatory roles across specific clinical contexts such as MDR infections, localized wound infections, pneumonia and osteomyelitis. As a primary core conclusion, the functional impact of these nanoscale vesicles is fundamentally dictated by the specific infectious microenvironment and the intricate cargo they transport. Within the restricted anatomical sites of pneumonia or the bone marrow niches of osteomyelitis, host-derived exosomes—particularly those originating from macrophages or MSCs—frequently activate protective host immunity. They achieve this by delivering AMPs or specific regulatory miRNAs that promote macrophage polarization toward a pro-inflammatory M1 phenotype, thereby enhancing phagocytic clearance. Conversely, bacterial pathogens exploit their own extracellular vesicles, such as OMVs, as potent long-range virulence delivery systems. In hypoxic wound beds and orthopedic tissues, these microbial vesicles carry an arsenal of effectors, including pore-forming toxins and lipopolysaccharides, to facilitate critical pathogenic processes such as biofilm formation, immune evasion and the horizontal transfer of antibiotic resistance genes. This intricate pathogen–host interaction heavily modulates the autophagy-apoptosis balance and drives the exosome-matrix metalloproteinase regulatory axis, accelerating severe tissue destruction and facilitating systemic pathogen spread.

Building upon these mechanistic insights, a second core conclusion underlines the immense potential of bioengineered exosomes as advanced, target-specific therapeutic vehicles capable of overcoming the limitations of conventional anti-infective strategies. By leveraging their low immunogenicity, exceptional biocompatibility and an innate capacity to penetrate dense bacterial biofilms, exosomes can be loaded with established antimicrobial agents to target recalcitrant infections. Encapsulating conventional antibiotics like polymyxin B or natural phytochemicals like curcumin within these nanocarriers achieves potent synergistic bactericidal effects against MDR strains, while packaging endogenous AMPs significantly enhances their intracellular delivery efficacy. Beyond small molecules, exosome-mediated transport offers a revolutionary platform for molecular interventions, including the delivery of gene-silencing siRNA targeting resistance determinants and CRISPR/Cas9 systems designed for the targeted knockout of essential bacterial virulence genes. To further enhance therapeutic precision, surface engineering methodologies—such as conjugating mannose moieties to target alveolar macrophages in pneumonia or anchoring pathogen-specific antibodies onto the exosome surface—enable infection site-specific enrichment, though fully maximizing their clinical efficacy necessitates a deeper understanding of their intracellular fate, uptake mechanisms and subcellular trafficking.

Complementing their therapeutic utility, a third core conclusion establishes that exosomes hold substantial diagnostic and prognostic value as accessible, compartment-specific reservoirs of clinical biomarkers. Bacterial-derived vesicles circulating in biofluids carry characteristic paternal antigens that allow for direct pathogen identification, while host-derived exosomal miRNAs provide real-time insights into host immune status and infection severity. To maximize diagnostic sensitivity and specificity, particularly for detecting elusive resistant strains, multiplexed assay platforms integrating techniques such as single particle interferometric reflectance imaging sensor with quantitative PCR are proving highly effective. Furthermore, the dynamic monitoring of exosome-associated immunosuppressive molecules offers immense promise for predicting the risk of chronicity in wound infections and osteomyelitis, as well as gauging real-time therapeutic responses. The clinical translation of these diagnostic applications is actively being explored in ongoing clinical trials, including plasma exosome miRNA profiling for sepsis diagnosis and urinary exosome antigen detection for noninvasive diagnosis of urinary tract infections.

**Figure 16:**
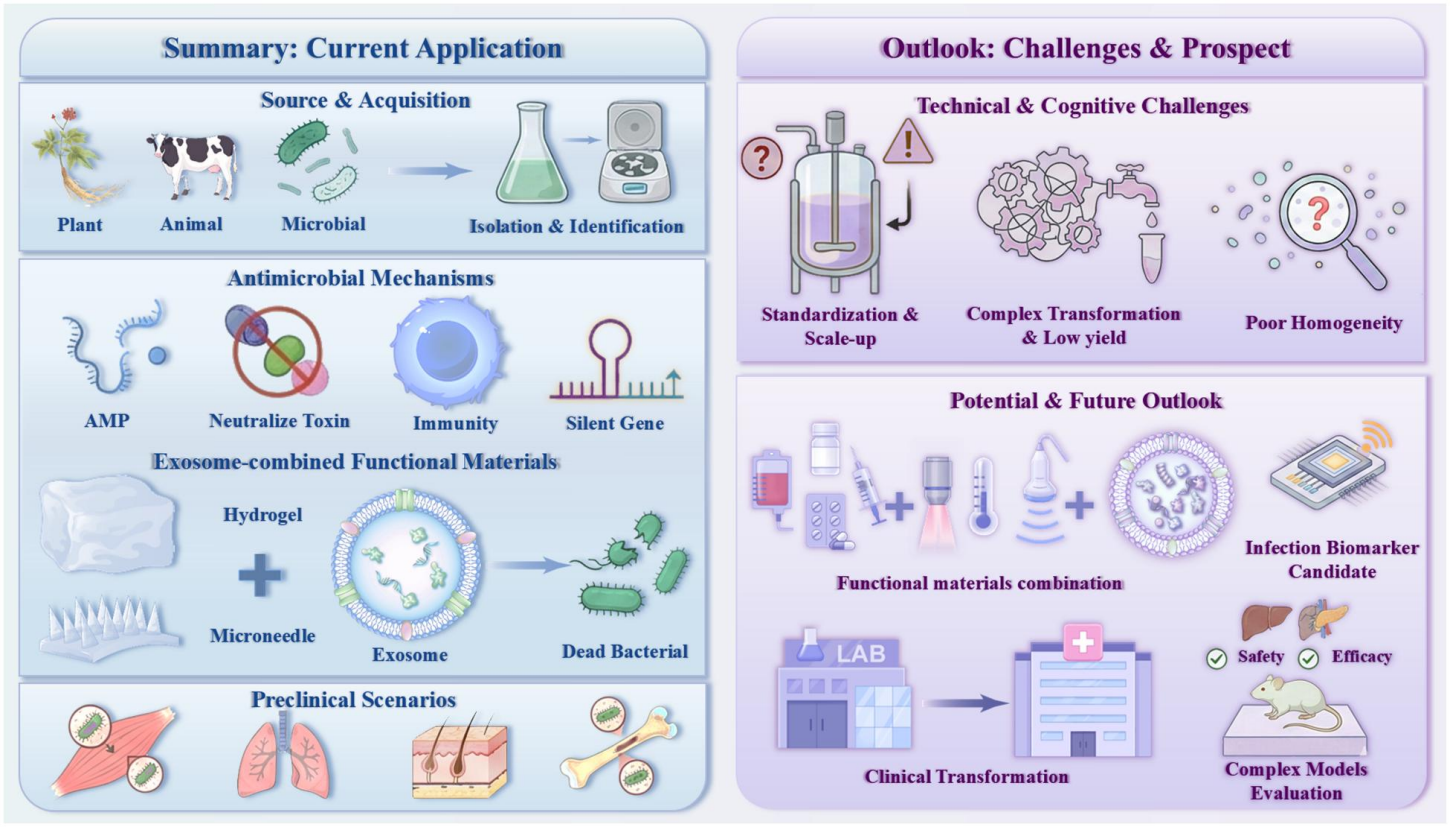
Summary of exosomes in the field of antibacterial therapy and future prospects. This section systematically reviews the sources, isolation and identification methods of exosomes, elaborates on their antibacterial mechanism and introduces the strategies for combining them with functional materials to achieve synergistic antibacterial effects. Currently, there are still insufficient understandings regarding the isolation and identification methods of exosomes. Regarding their future applications, such as biomarkers, combinations with photothermal materials and clinical translation, this article further presents prospects.

Despite these significant advancements, substantial challenges surrounding isolation purity, scalable production and unresolved *in vivo* pharmacokinetics continue to impede the immediate clinical translation of exosome-based technologies. Crucially, over the next 3–5 years, the most critical and feasible breakthrough directions in this field are projected to converge on three major dimensions. First, the development of standardized, automated microfluidic-based isolation platforms is expected to achieve high-throughput, high-purity separation of host-derived exosomes from co-existing bacterial OMVs within complex patient matrices. Second, the clinical translation of advanced hybrid biomaterials—such as engineering exosome-photothermal or bio-responsive hydrogel delivery systems—will provide a feasible breakthrough for the localized, sustained eradication of deep-seated biofilms in chronic wound infections and recalcitrant osteomyelitis. Third, the establishment of regulatory-compliant, off-the-shelf engineered vesicles derived from standardized, immortalized cell lines will accelerate comprehensive safety and efficacy assessments in large-animal models of severe MDR sepsis. Collectively, addressing these specific technological hurdles and pursuing these strategic research directions will successfully bridge the translational gap, moving exosome-based technologies from promising benchtop concepts toward tangible precision diagnostics and targeted therapies for refractory bacterial infections (Figure 16).
